# Stigmasterol-Mediated Targeting of Rho-Associated Coiled-Coil Protein Kinase 1 Ameliorates Diabetic Kidney Disease and Attenuates Renal Tubular Lipid Deposition

**DOI:** 10.34133/bmr.0389

**Published:** 2026-07-17

**Authors:** Yuchi Chen, Xinyao Xu, Ningning Yuan, Yangtian Yan, Yuxin Ye, Zhuoen He, Jinyue He, Chi Zhang, Hao Wang, Haitao Yuan, Jianxin Diao, Wei Xiao

**Affiliations:** ^1^ School of Traditional Chinese Medicine, Southern Medical University, Guangzhou, Guangdong 510515, China.; ^2^ College of Life Science, Zhuhai College of Science and Technology, Zhuhai 519090, China.; ^3^ Center for Drug Research and Development, Guangdong Provincial Key Laboratory of Pharmaceutical Preparations Research and Evaluation, Guangdong Pharmaceutical University, Guangzhou 510006, China.; ^4^ Key Laboratory of Glucolipid Metabolic Disorder, Ministry of Education, Guangdong Pharmaceutical University, Guangzhou, Guangdong 510006, China.; ^5^ Guangdong Provincial Key Laboratory of Autophagy and Major Chronic Non-communicable Diseases, Affiliated Hospital of Guangdong Medical University, Zhanjiang, Guangdong 524001, China.

## Abstract

Diabetic kidney disease (DKD) is identified as the major contributor to the development of end-stage renal disease, with its clinical incidence increasing. Emerging studies link DKD closely to renal lipid deposition, tubular injury, and glomerulosclerosis—pathological processes driven by renal lipid metabolism disorders that ultimately induce renal fibrosis. However, targeted therapeutics for renal lipid deposition are scarce. This study fills this research gap: first, clinical database analyses identified a positive correlation between up-regulated rho-associated coiled-coil protein kinase 1 (ROCK1) expression in renal tubules and progressive renal function deterioration in DKD patients, a finding recapitulated in DKD mouse models, which also exhibited renal tubular ROCK1 up-regulation and concomitant lipid accumulation; second, molecular docking, surface plasmon resonance, and cellular thermal shift assay confirm that the natural molecule stigmasterol (ST) binds to ROCK1 and inhibits its expression with a dose-dependent trend; and, third, in vivo and in vitro experiments demonstrate that ST alleviates lipid accumulation, mitochondrial damage, and renal fibrosis in DKD via the ROCK1/p38 mitogen-activated protein kinase/peroxisome proliferator-activated receptor α axis. In conclusion, ST exerts direct renoprotective effects by regulating the ROCK1 pathway to improve renal lipid metabolism, reduce mitochondrial damage, and inhibit fibrosis, highlighting its potential as a novel ROCK1 inhibitor. This study identifies ST as a candidate for targeted intervention in DKD-related lipid metabolism, validates ROCK1 as a therapeutic target, provides an experimental basis for the DKD treatment strategy of “targeting ROCK1 to synergistically improve lipid metabolism and mitochondrial function”, and opens new avenues for natural products in metabolism-related nephropathies.

## Introduction

Diabetic kidney disease (DKD), a major microvascular complication associated with diabetes, exhibits a high incidence among diabetic patients. Approximately 30% of patients with type 1 diabetes and 40% of those with type 2 diabetes may progress to DKD. Currently, it has become the leading cause of end-stage renal disease (ESRD) in China [1]. Clinically, DKD is characterized by a persistent increase in urinary albumin excretion or a decrease in glomerular filtration rate [2], and its therapeutic options remain limited. At present, clinical therapeutic strategies are mainly limited to symptomatic interventions such as glycemic modulation, blood pressure regulation, lipid regulation, and the mitigation of proteinuria, supplemented by lifestyle interventions including dietary adjustment and regular exercise.

However, existing treatment strategies have major limitations: they can only delay the progression of DKD to a certain extent and fail to achieve the reversal and repair of renal function. When patients progress to the ESRD stage, they must rely on alternative treatments such as kidney transplantation or maintenance hemodialysis to sustain life. With the continuous rise in the morbidity and mortality of DKD, it not only severely impairs patients’ quality of life but also poses a major challenge to human health and public health security. Accordingly, there is an urgent clinical demand in modern medicine for the development of novel therapeutic regimens targeting DKD through exploratory research.

DKD pathogenesis is driven by a multitude of pathogenic factors, among which are dyslipidemia, mitochondrial dysfunction, oxidative stress, and fibrosis, with concomitant characteristic renal lesions including glomerular podocyte loss, mesangial hyperplasia, renal tubular epithelial cell injury, and renal tubulointerstitial fibrosis [3–5]. Renal tubular cells, as key effector cells responsible for renal reabsorption function, play a crucial role in maintaining the homeostasis of renal physiological functions [6]. The kidney is also an important organ for lipid filtration and metabolism [7], and dyslipidemia has additionally been identified as one of the pivotal risk factors in the pathogenesis of DKD. During the pathological progression of DKD, renal lipid deposition is relatively common. Abnormally elevated blood lipid levels can damage the renal vascular structure and the integrity of the endothelial barrier [8]. Based on this, the Kidney Disease Outcomes Quality Initiative guidelines explicitly recommend that DKD patients receive statin therapy for lipid regulation [9].

Mitochondria generate large amounts of reactive oxygen species (ROS) through the respiratory chain, making them the primary endogenous source of ROS in the body. In patients with DKD, persistent disturbances in glucose and lipid metabolism lead to abnormal accumulation of free lipids in renal tubular epithelial cells. Excessive lipid toxicity directly attacks the mitochondrial structure, causing disruption of mitochondrial cristae and mitochondrial dysfunction. Damaged mitochondria further induce disturbances in the electron transport chain and a surge in ROS, persistently activating oxidative stress responses. Excessive oxidative stress, in turn, exacerbates lipid metabolic disorders and mitochondrial injury, creating a vicious cycle that ultimately drives tubulointerstitial damage, collagen deposition, and progression of DKD.

Rho-associated coiled-coil protein kinase (ROCK) belongs to the serine/threonine protein kinase family and primarily regulates biological processes such as cell contraction, migration, and proliferation [10]. The ROCK family consists of 2 isozymes: ROCK1 (mainly expressed in liver, kidney, and lung tissues) [11,12] and ROCK2. Numerous studies have established that excessive activation of the ROCK signaling pathway substantially promotes the development of glomerular fibrosis and podocyte injury during renal pathological processes [13]. In addition, as a core regulatory factor of the hepatic lipid metabolic network, ROCK1 plays a critical role in modulating lipid homeostasis. Studies have confirmed that inhibition of ROCK1 activity can activate the AMPK signaling pathway, down-regulate the expression of lipid-metabolism-related transcription factors, restore AMPK phosphorylation-mediated fatty acid metabolism function in mesangial cells, enhance glomerular fatty acid oxidation (FAO) levels [14], and improve mitochondrial dysfunction in renal tubular cells and podocytes [15,16]. These findings highlight the critical role of ROCK1 in the regulation of DKD-related lipid disorders. On the other hand, enhanced ROCK activity can also exacerbate renal injury by promoting oxidative stress, inducing sodium retention, and increasing vascular tension [17]. Meanwhile, it impairs the protein reabsorption function of proximal tubular cells, leading to further deterioration of proteinuria [18].

Currently, clinical management strategies for DKD remain limited, and the overall therapeutic efficacy has not yet reached the desired level. As a class of natural bioactive compounds, phytosterols have been widely recognized for their beneficial effects on human health [19]. Studies have reported that they can exert lipid-lowering effects by reducing intestinal cholesterol absorption and decreasing low-density lipoprotein cholesterol (LDL-C) levels [20] and have been approved by the US Food and Drug Administration as cholesterol-lowering agents. Stigmasterol (ST) is a natural phytosterol abundant in beans, nuts, and medicinal plants, with diverse pharmacological activities. Firstly, in streptozotocin (STZ)-induced DKD model rats, ST can improve superoxide dismutase activity and glutathione levels and alleviate lipid peroxidation, thereby exerting antioxidant protective effects [21]. Secondly, ST can reduce the degree of renal fibrosis by regulating mitophagy processes and the intestinal flora structure [22]. Thirdly, in terms of lipid metabolism regulation, ST can promote intestinal cholesterol excretion and exert its effects by competitively inhibiting intestinal cholesterol absorption and improving hepatic lipid metabolism disorders. In addition, several studies have suggested that natural extracts containing ST can help alleviate symptoms of type 2 diabetes by inhibiting the activity of α-glucosidase [23] and α-amylase [24]. Based on the aforementioned multiple pharmacological activities, as a natural drug, ST exhibits substantial developmental potential for the preventive and therapeutic management of DKD. However, current research on the mechanism of ST in DKD remains insufficiently in-depth, and there is an urgent need to further systematically explore its targets and regulatory networks to provide a theoretical foundation for the clinical translation, development, and utilization of ST. Meanwhile, it is necessary to strengthen interdisciplinary cooperation, integrate technical approaches from multiple fields, and promote the coordinated advancement of ST-related basic and applied research.

To further explore how the core regulatory pathways of renal lipid metabolism disorders (e.g., the ROCK1–peroxisome proliferator-activated receptor α [PPARα] axis) drive renal fibrosis in DKD through “cell-specific mechanisms”, we focused on the following pathological cascade: lipid accumulation in renal tubular epithelial cells → fatty acid metabolism disorders and mitochondrial damage → increased inflammation and oxidative stress. The key question is, can natural bioactive molecules simultaneously improve renal lipid metabolism and mitochondrial function by targeting this pathway, thereby breaking the vicious cycle of “lipid disorder–renal injury”? This strategy has become a potential candidate for the precise intervention of DKD-related renal fibrosis.

**Fig. 1.**
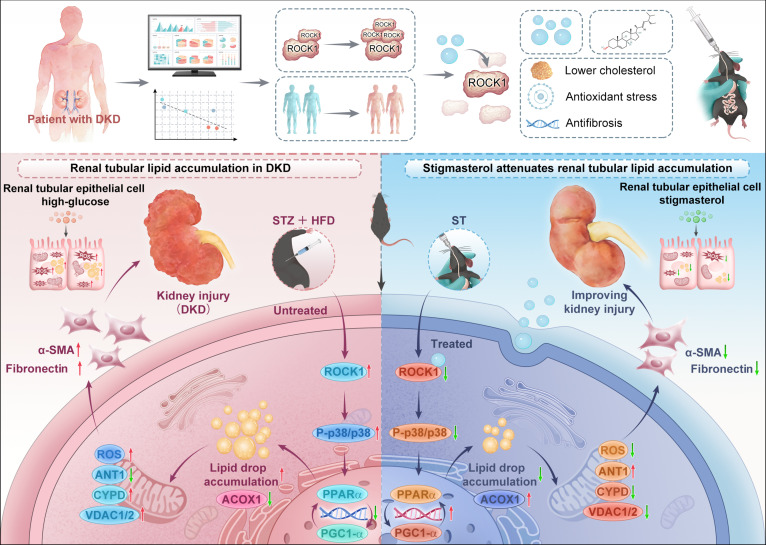
Schematic illustration of stigmasterol targeting the rho-associated coiled-coil protein kinase 1 (ROCK1)/p38 mitogen-activated protein kinase (MAPK)/peroxisome proliferator-activated receptor α (PPARα) axis for lipid metabolism regulation in diabetic kidney disease (DKD).

Based on this premise, the present study obtained the following key findings: (a) The expression of ROCK1 was markedly up-regulated in DKD patients and mice, indicating that ROCK1 is a potential regulatory target for DKD. (b) Molecular docking, surface plasmon resonance, and cellular thermal shift assay experiments confirmed that ST can directly bind to ROCK1 with high affinity. This binding notably enhanced the thermal stability of ROCK1 and down-regulated its protein expression in a dose-proportional manner. (c) In vivo experiments illustrated that ST intervention substantially reduced serum creatinine (SCr) and blood urea nitrogen (BUN) levels in DKD mice, decreased collagen deposition and lipid accumulation in the renal tubular region, reduced ROS accumulation, alleviated podocyte injury, and improved renal tubular mitochondrial swelling, crista shortening, and crista loss. (d) In vitro cell experiments further verified that ST effectively inhibited lipid droplet formation in high-glucose (HG)-induced HK-2 cells, up-regulated the expression of FAO-related genes, and regulated the expression of mitochondrial-damage-related genes. Mechanistically, we confirmed that ST exerted dual regulatory effects by inhibiting ROCK1: on one hand, it down-regulated the phosphorylation of downstream p38 mitogen-activated protein kinase (p38 MAPK) and up-regulated the expression of PPARα, thereby improving lipid metabolism disorders; on the other hand, it alleviated hyperglycemia-induced mitochondrial structural damage and dysfunction. These 2 pathways collectively inhibit renal tubular fibrosis, as reflected by the down-regulation of α-smooth muscle actin (α-SMA) and fibronectin (Fig. 1). In conclusion, this study clarifies the mechanism of ST by targeting ROCK1, providing an ideal candidate molecule for the clinical development of “safe ROCK1-targeted therapeutic drugs” and offering a broad perspective for the research on “metabolism-related nephropathy” and “natural product mechanism exploration”.

## Materials and Methods

### Clinical analysis

We utilized the Nephroseq v5 database (http://v5.nephroseq.org)—a comprehensive resource for analyzing gene expression–clinical feature correlations in renal diseases—to perform data mining for exploring the relationship between ROCK1 expression and DKD-relevant clinical parameters. DKD samples’ data are shown in Table S1.

### Cell culture and chemicals

Human renal proximal tubular epithelial (HK-2) cells were purchased from Procell Life Science & Technology Co., Ltd. (Wuhan, China), authenticated by short tandem repeat profiling (authenticated on 2024 April 28), and confirmed to be mycoplasma-free. Cell cultures were maintained in Dulbecco’s modified Eagle medium/F12 medium containing 10% fetal bovine serum and 1% penicillin–streptomycin (referred to as complete medium hereafter) under a humidified 5% CO_2_ atmosphere at 37 °C. Cells were passaged using trypsin digestion when reaching 70% to 80% confluence and cryopreserved in freezing medium at −80 °C for long-term storage. For experiments, cells at 80% confluence were divided into 4 groups: control (5.5 mM glucose), HG (35 mM glucose), and 2 treatment groups (HG + 5 or 10 μM ST). ST, obtained with a purity of over 95% (Cat. No. AB0177, Chengdu, China), was dissolved in dimethyl sulfoxide (DMSO) and applied to the culture system at concentrations of 5 or 10 μM. Cells were incubated under these conditions for 24 h prior to subsequent analyses.

### siRNA transfection

For gene knockdown experiments, HK-2 cells at 70% confluency were transfected with small interfering RNA (siRNA) by means of Lipofectamine 3000. Briefly, siRNA and Lipofectamine 3000 were each diluted in Opti-MEM medium, followed by mixing and incubation to form transfection complexes. The complexes were added to cells in serum-free medium for 6 to 8 h, after which the medium was replaced with complete medium. Transfected cells were harvested 24 h posttransfection for quantitative real-time polymerase chain reaction (qPCR) analysis or 48 h posttransfection for Western blotting (WB) to assess knockdown efficiency and downstream effects. siRNA and qPCR primer sequences are shown in Tables S2 and S3, respectively.

### Animal model and experimental design

#### Animal housing and initial procedures

Male C57BL/6J mice (6 weeks old) were purchased from the Laboratory Animal Center of Southern Medical University. The animals were housed in a specific-pathogen-free facility under controlled conditions (22 °C, 12-h light/dark cycle). All experimental procedures were approved by the Animal Ethics Committee of Southern Medical University (approval number: L2022321; date of resolution: 2023 February 5) according to the Guide for the Care and Use of Laboratory Animals of the National Institutes of Health.

#### DKD model induction and drug delivery interventions

Twenty-four male C57BL/6J mice (6 weeks old) were acclimatized for 7 d before randomization into control (*n* = 6, standard diet) and model (*n* = 18, high-fat diet [HFD]) groups. After 4 weeks, the model group received STZ (50 mg/kg/d, pH 4.5 citrate buffer, 5 consecutive days) with 12-h fasting. DKD was confirmed by fasting blood glucose (FBG) ≥11.1 mmol/l (on 3 consecutive measurements) and the presence of 24-h proteinuria on day 7 post-STZ injection. Using a random number table, successful DKD models were randomly divided into the DKD model (vehicle control), ST-low (50 mg/kg/d), and ST-high (100 mg/kg/d) groups (*n* = 6 each) [25,26]. All groups received daily oral gavage of 0.5% sodium carboxymethyl cellulose suspension containing ST or vehicle (normal saline for controls) between 9:00 and 11:00 AM for 5 consecutive weeks. Body weight and FBG were monitored weekly. Upon completion of the intervention period, 24-h urinary protein levels were quantified.

### Renal tissue staining

Renal tissues were fixed in 4% paraformaldehyde and embedded for sectioning at 4-μm thickness. Sections underwent deparaffinization and rehydration for histological staining procedures including hematoxylin–eosin (H&E), periodic acid–Schiff (PAS), and Masson staining (all kits from Beijing Solarbio Science & Technology Co., Ltd.) to evaluate the renal pathological morphology, glycogen accumulation, and collagen deposition and to analyze the degree of damage of DKD and the therapeutic effects of ST. Images were captured using an OLYMPUS microscope with random field selection for unbiased analysis. Image Pro Plus 6.0 was used for quantitative analysis, and data are presented as the positive area percentage.

### Biochemical assays

Commercial assay kits (Nanjing Jiancheng Bioengineering Institute, China) were utilized to determine levels of SCr (C011-2-1), BUN (C013-1-1), 24-h urinary protein (C035-1-1), total cholesterol (TC; A111-1-1), triglycerides (TG; A110-1-1), and LDL-C (A113-1-1) following the manufacturers’ instructions.

### Oil Red O staining

Renal tissues were cryosectioned at 10 μm, fixed in 4% paraformaldehyde for 10 min, and stained with Oil Red O solution (60% isopropanol for 20 s, staining for 30 min). HK-2 cells were fixed and stained similarly (50-min staining). Both were counterstained with hematoxylin and mounted with glycerin gelatin.

### Transmission electron microscopy observation

Following rapid excision, renal cortex tissues were fixed in 2.5% glutaraldehyde, rinsed thrice with phosphate-buffered saline, and subjected to postfixation in 1% osmium tetroxide for 2 h. After dehydration via a graded ethanol gradient, samples were embedded in epoxy resin and sectioned into ultrathin slices. The sections were stained with uranyl acetate and lead citrate solutions, mounted on sample holders, and imaged under a transmission electron microscope after fine-tuning imaging parameters; representative images were acquired for analytical purposes.

### Immunohistochemistry

Paraffin sections (4 μm) underwent antigen retrieval in citrate buffer (pH 6.0, G1201-1L, Servicebio, Wuhan, China), peroxidase blocking, and 1-h blocking with 5% goat serum (ZLI-9018, ZSGB-BIO, Beijing, China). Primary antibodies against ROCK1 (1:200, 21850-1-AP, Proteintech, Wuhan, China), PPARα (1:200, ab61182, Abcam, UK), P-p38 (1:100, 4511T, Cell Signaling Technology, USA), p38 (1:100, 14064-1-AP, Proteintech, Wuhan, China), fibronectin (1:200, 15613-1-AP, Proteintech, Wuhan, China), and α-SMA (1:200, 67735-1-Ig, Proteintech, Wuhan, China) were incubated overnight at 4 °C, followed by horseradish peroxidase (HRP)-conjugated secondary antibodies and 3,3′-diaminobenzidine (AR0009, BOSTER, Wuhan, China) development.

### Western blotting

HK-2 cells or renal tissues were lysed in radioimmunoprecipitation assay buffer (R0020, Solarbio, Beijing, China) supplemented with protease and phosphatase inhibitors. The total protein concentration was quantified using the BCA Protein Assay Kit (PC0020, Solarbio, Beijing, China). Denatured protein samples (30 μg per lane) were resolved by 10% sodium dodecyl sulfate–polyacrylamide gel electrophoresis and electrophoretically transferred to polyvinylidene difluoride membranes (ISEQ00010, Merck, Germany). Membranes were blocked with 5% nonfat milk in tris-buffered saline containing 0.1% Tween-20 (TBST) for 2 h at room temperature, followed by overnight incubation at 4 °C with primary antibodies under gentle agitation. Membranes were washed 3 times with TBST (10 min per wash) and then incubated with HRP-conjugated secondary antibodies (1:10,000, Proteintech, Wuhan, China) for 2 h at 4 °C under shaking conditions; this was followed by a further three 10-min TBST washes. An enhanced chemiluminescence reagent was used to visualize protein bands on a FluorChem E imaging system (ProteinSimple, San Francisco, CA, USA). The following primary antibodies were used: ROCK1 (1:1,000, 21850-1-AP, Proteintech, Wuhan, China), PPARα (1:1,000, ab61182, Abcam, UK), p38 MAPK (1:2,000, 14064-1-AP, Proteintech, Wuhan, China), phospho-p38 MAPK (Thr180/Tyr182) (D3F9) XP Rabbit mAb (1:1,000, 4511T, Cell Signaling Technology, USA), fibronectin (1:2,000, 15613-1-AP, Proteintech, Wuhan, China), α-SMA (1:20,000, 67735-1-Ig, Proteintech, Wuhan, China), β-tubulin (1:1,000, AF7011, Affinity Biosciences, Jiangsu, China), and glyceraldehyde-3-phosphate dehydrogenase (1:8,000, 60004-1-Ig, Proteintech, Wuhan, China).

### Statistical analysis

Statistical analysis was performed on data (mean ± standard error of the mean) via GraphPad Prism 9.0: Student *t* test for 2-group comparisons and one-way analysis of variance for multiple-group comparisons (*P* < 0.05, significant). All experimental assays were repeated a minimum of 3 times.

## Results

### ROCK1 is highly expressed in DKD patients and mice

**Fig. 2.**
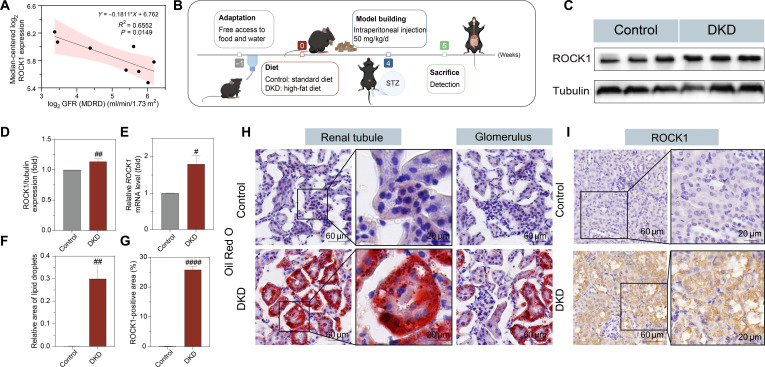
Rho-associated coiled-coil protein kinase 1 (ROCK1) is highly expressed in diabetic kidney disease (DKD) patients and mice. (A) Correlation analysis of ROCK1 expression in renal tubules with renal function. (B) Schematic diagram of an animal experiment. (C) Protein blotting assay for the expression of ROCK1. (D) Quantitative analysis of ROCK1 by protein blotting. (E) The messenger RNA (mRNA) levels of *ROCK1* were analyzed using quantitative real-time polymerase chain reaction (qPCR). (F) Quantitative map of Oil Red O in kidney tissue. (G) Quantitative analysis of immunohistochemical (IHC) staining for ROCK1 in mouse kidney tissues. (H) Oil Red O staining of mouse kidney tissue (×200 magnification, bar = 60 μm/20 μm). (I) IHC staining of ROCK1 in mouse kidney tissues (×200 magnification, bar = 60 μm/20 μm). The data are presented as mean ± standard error of the mean (SEM) (*n* = 3/group). ^#^*P* < 0.05, ^##^*P* < 0.01, ^###^*P* < 0.001, and ^####^*P* < 0.0001, DKD vs. control; ns, not significant.

As a serine/threonine protein kinase, ROCK1 is tightly correlated with renal mesangial cell proliferation and renal fibrosis. As shown in Fig. 2A, ROCK1 expression was markedly elevated in the renal tubules of patients with DKD, and this up-regulation exhibited a strong negative correlation with renal function (*R*^2^ = 0.6552, *P* = 0.0149). These results identify ROCK1 as a pivotal therapeutic target driving the progression of DKD, thereby providing a robust experimental rationale for subsequent in vivo investigations. We established a DKD mouse model using STZ combined with an HFD (Fig. 2B). To clarify the expression profile of ROCK1 in DKD renal tissues, we quantified the protein and messenger RNA (mRNA) levels of ROCK1 in the renal cortex using WB and qPCR (Fig. 2C to E). Experimental results established that ROCK1 expression was notably higher in the renal tissues of the DKD model mice compared to that in normal healthy controls. Additionally, Oil Red O staining indicated prominent lipid accumulation in the renal tubular region (Fig. 2F and H)—a pathological change that can trigger oxidative stress, inflammatory activation, and apoptosis. These events collectively cause severe damage to renal tubular epithelial cells and glomerular podocytes, ultimately leading to proteinuria and reduced glomerular filtration rate in mice, which accelerates the pathological progression of DKD [27,28].

Further immunohistochemical (IHC) staining of mouse kidney sections confirmed high ROCK1 expression in the renal tubular region (Fig. 2G and I). In summary, ROCK1 is overexpressed in DKD; given its key regulatory role in lipid metabolism, we hypothesize that ROCK1 may be associated with renal tubular lipid accumulation in DKD. Phytosterols have demonstrated favorable therapeutic efficacy in the management of DKD. Thus, we hypothesize that ROCK1 may function as a key potential therapeutic target for natural molecules in the targeted therapy of DKD. The development of novel ROCK1 inhibitors to target the ROCK1 pathway—thereby improving renal lipid metabolism, alleviating mitochondrial damage, and inhibiting renal fibrosis to achieve renal protection—will emerge as a highly promising strategy in the current clinical management of DKD.

### Molecular docking screened ST as a potential inhibitor of ROCK1

**Fig. 3.**
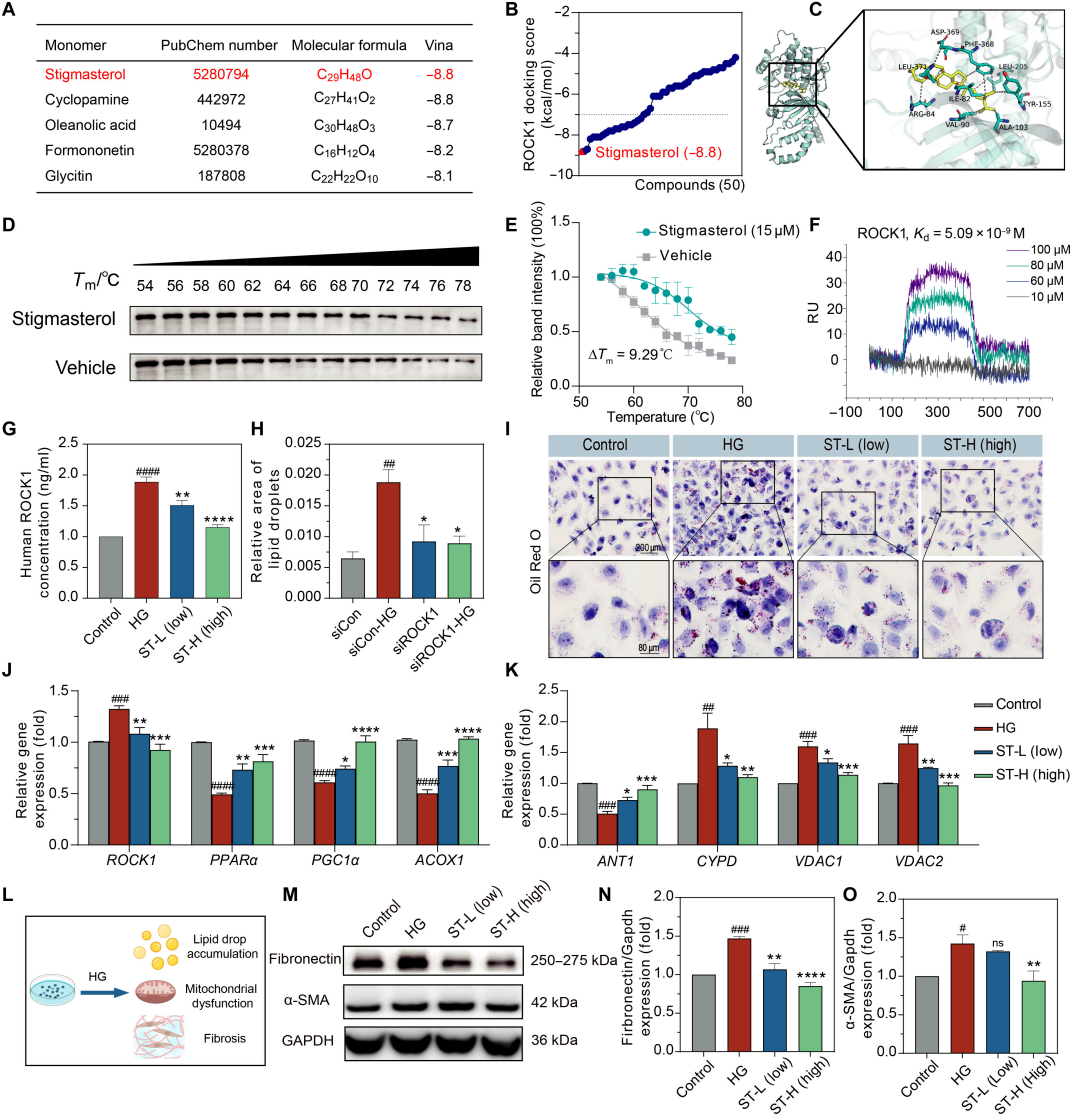
Molecular docking screened stigmasterol (ST) as a potential inhibitor of rho-associated coiled-coil protein kinase 1 (ROCK1). (A) The molecular docking table of ROCK1. (B) Docking scores of 50 natural compounds with ROCK1. (C) Binding mode of stigmasterol. (D) Western blot results from cellular thermal shift assay (CETSA). (E) CETSA thermogram showing ROCK1 thermal stability changes induced by ST. (F) Plot of the course of change in response units (RU) of ST and ROCK1. (G) Effect of ST on ROCK1 enzyme-linked immunosorbent assay (ELISA). (H) Quantitative analysis of Oil Red O staining. (I) Oil Red O staining of HK-2 cells (×200 magnification, bar = 200 μm/80 μm). (J) Messenger RNA (mRNA) levels of *ROCK1*, *PPARα*, *PGC1α*, and *ACOX1*. (K) mRNA levels of *ANT1*, *CYPD*, *VDAC1*, and *VDAC2*. (L) Mechanism of the cell model. (M) Detection of α-smooth muscle actin (α-SMA) and fibronectin protein in HK-2 cells by protein blotting. (N) Quantitative analysis of fibronectin by protein blotting. (O) Quantitative analysis of α-SMA by protein blotting. The data are presented as mean ± standard error of the mean (SEM) (*n* = 3/group). ^#^*P* < 0.05, ^##^*P* < 0.01, ^###^*P* < 0.001, and ^####^*P* < 0.0001, high-glucose (HG) vs. control; *P < 0.05, **P < 0.01, ***P < 0.001, and ****P < 0.0001, HG vs. ST; ns, not significant.

To identify natural compounds that can target ROCK1 and act as ROCK1 inhibitors, we first performed molecular docking of 50 compounds from a natural compound library with ROCK1 (Table S4). A binding energy of <−6 kcal/mol was defined as a strong binding affinity. Results showed that ST exhibited the strongest binding to ROCK1, with a binding affinity of −8.8 kcal/mol (Fig. 3A and B). Further analysis of the binding mode revealed that ST formed 3 conventional hydrogen bonds with the ASP-369, TYR-155, and ARG-84 residues of ROCK1 (the receptor), as well as 6 hydrophobic interactions with residues including PHE-368 and LEU-205 (Fig. 3C).

The molecular structure of ST is presented in Fig. S1. To verify the binding stability between ST and ROCK1, we assessed ROCK1 protein thermal stability. Exogenous ST addition notably enhanced ROCK1 thermal stability, indicating the formation of a stable ST–ROCK1 complex. Quantitative analysis showed that the melting temperature (*T*_m_) of ROCK1 in the ST-treated group was 9.29 °C higher than that in the DMSO control group (Fig. 3D and E). Surface plasmon resonance experiments further confirmed the specific binding of ST to ROCK1: the binding signal was evident and dose dependent, with a binding constant (*K*_d_) of 5.09 × 10^−9^ M (Fig. 3F). Together, these results demonstrate a direct interaction between ST and ROCK1; however, the targeted inhibitory effect of ST on ROCK1 required further verification via enzyme-linked immunosorbent assay. Results demonstrated that ROCK1 expression was markedly increased in the HG model group, reaching 1.88-fold that of the normal control group. With increasing ST treatment concentration, ROCK1 expression decreased in a concentration-dependent manner (Fig. 3G); ROCK1 is regulated by the ubiquitin-proteasome system. ST modulates E3–substrate interactions (e.g., inhibiting ORP5 ubiquitination [29]). Thus, ST binding to ROCK1 may recruit an E3 ligase to ROCK1, increasing its ubiquitination and degradation. Moreover, ST may induce a conformational change in ROCK1, exposing a hidden degron for E3 recognition, as seen with BACH1 [30]. In summary, ST dose-dependently inhibits ROCK1 expression.

Network pharmacology was employed to preliminarily identify the pharmacological targets of ST in the treatment of DKD. Based on the Metascape platform, enrichment analysis of overlapping genes was conducted by integrating Gene Ontology and Kyoto Encyclopedia of Genes and Genomes (KEGG) annotations, with statistical significance assessed by the *P* value. For each annotation category, the top 10 enriched terms were ranked in ascending order of log*P* values and visualized as histograms (Fig. S2A). Additionally, the top 12 KEGG enrichment results were presented as bubble plots, also sorted by ascending log*P* values (Fig. S2B). Notably, these enriched pathways included steroid hormone biosynthesis, the PPAR signaling pathway, arachidonic acid metabolism, and nonalcoholic fatty liver disease—all of which are closely associated with lipid metabolism. Search Tool for the Retrieval of Interacting Genes/Proteins (STRING) analysis was further used to perform overlapping analysis of ST target genes and DKD-related target genes, and a protein–protein interaction network was constructed to characterize the connectivity of core targets (Fig. S2C). Through this analysis, we identified the core targets CYP19A1, HMGCR, ESR1, PPARG, CYP17A1, ESR2, PPARA, and HSD11B1, among which HMGCR, PPARG, ESR2, PPARA, and HSD11B1 are tightly linked to lipid metabolism. Collectively, these findings indicate that ST may ameliorate the progression of DKD by regulating lipid metabolic homeostasis via lipid-metabolism-associated targets and signaling pathways.

Given that renal tubular cells preferentially use fatty acids for energy production, defects in their fatty acid metabolism are closely associated with renal injury. Thus, renal tubules serve as an ideal model for investigating FAO and related metabolic disorders [31]. Based on the above findings, we selected human proximal tubular epithelial (HK-2) cells for in vitro studies. The mycoplasma-negative staining results of HK-2 cells are presented in Fig. S3. Oil Red O staining was performed following HG-induced modeling, and results showed a marked increase in the area of Oil Red O-positive staining in the HG group—indicating that an HG environment induces lipid accumulation in HK-2 cells (Fig. 3H and I).

Alleviating this lipid accumulation requires promoting mitochondrially mediated FAO, which is also the primary energy-producing pathway in proximal renal tubular epithelial cells [32]. Peroxisome proliferator-activated receptor gamma coactivator 1α (PGC1α) acts as a coactivator of PPARα; they synergistically regulate biological processes, including energy metabolism, FAO—with acyl-CoA oxidase 1 (ACOX1) being a key enzyme promoting fatty acid β-oxidation—and mitochondrial function (e.g., *ANT1*, *VDAC1*, *VDAC2*, and *CYPD* are key genes regulating mitochondrial damage).

To explore this, we quantified the mRNA expression of lipid-metabolism-related key genes (*PPARα*, *PGC1α*, and *ACOX1*) via qPCR. Results revealed that the mRNA expression of these genes was markedly down-regulated in the HG group (Fig. 3J), indicating that an HG environment inhibits FAO in HK-2 cells. This inhibitory effect was mitigated following ST intervention. Additionally, in the HG group, the mRNA expression of *ANT1* (linked to energy metabolism) was strongly down-regulated, while the expression of *CYPD* (a gene regulating the mitochondrial permeability transition pore) and *VDAC1*/*VDAC2* (genes positively correlated with mitochondrial membrane permeability) was notably up-regulated. ST intervention reversed the abnormal expression of these genes (Fig. 3K).

WB results further showed that protein expression of α-SMA and fibronectin was increased in the HG group, indicating that HG induces HK-2 cell fibrosis. After ST intervention, the levels of these fibrosis-related proteins were noticeably down-regulated, demonstrating that ST can alleviate the fibrotic process in HK-2 cells (Fig. 3M to O). In conclusion, ST acts as a potential ROCK1 inhibitor, as it mitigates HG-induced lipid accumulation in HK-2 cells and ameliorates associated mitochondrial damage and fibrosis (Fig. 3L). These results provide direct and reliable cytological evidence for subsequent in vivo experiments aimed at verifying that “ST delays DKD progression by targeting ROCK1”.

### ST attenuates lipid accumulation in HK-2 cells via the ROCK1/p38 MAPK/PPARα pathway

p38 MAPK is a core molecule in the stress-responsive signaling pathway, mediating cellular responses to environmental stress, inflammatory stimuli, and various cytokines. Upon activation, this kinase phosphorylates a range of downstream target molecules—including transcription factors and cytoskeleton-associated proteins—thereby regulating key biological processes such as gene expression, cell cycle progression, apoptosis, and inflammatory responses.

A complex regulatory cross talk exists between p38 MAPK and PPARα, and this molecular interaction is critical for modulating fatty acid metabolism and inflammatory signaling responses. Multiple cell models have confirmed that p38 MAPK can negatively regulate PPARα transcriptional activity via phosphorylation, ultimately inhibiting FAO [33].

**Fig. 4.**
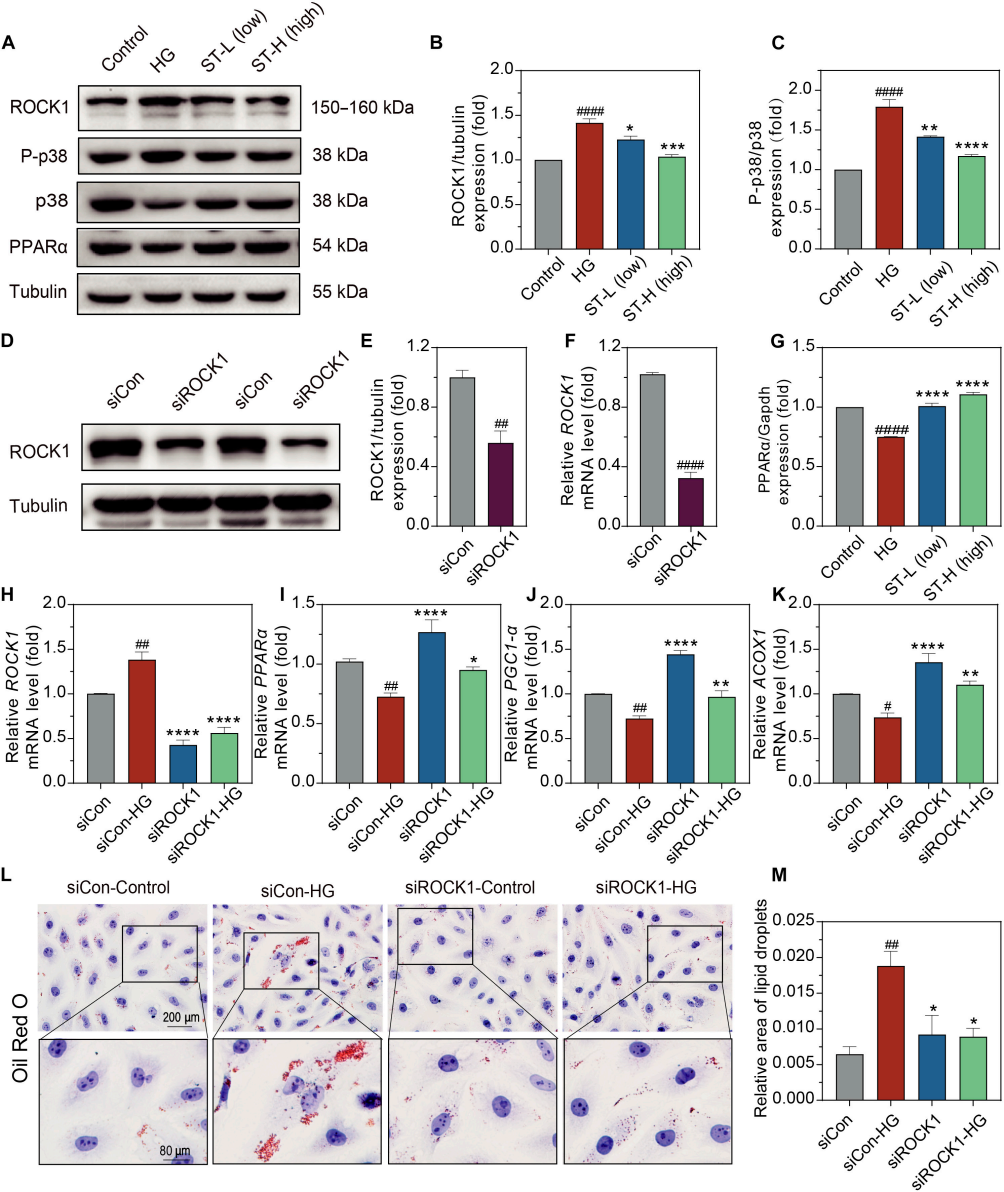
Stigmasterol (ST) attenuates lipid accumulation in HK-2 cells via the rho-associated coiled-coil protein kinase 1 (ROCK1)/p38 mitogen-activated protein kinase (MAPK)/peroxisome proliferator-activated receptor α (PPARα) pathway. (A) Protein blotting assay for the expression of ROCK1, P-p38, p38, and PPARα in HK-2 cells. (B, C, and G) Quantitative analysis of ROCK1, P-p38/p38, and PPARα by protein blotting. (D to F) HK-2 cells were transfected with either a control small interfering RNA (siRNA) or ROCK1 siRNA. The messenger RNA (mRNA) levels of *ROCK1* were analyzed using quantitative real-time polymerase chain reaction (qPCR). The protein levels of ROCK1 were determined by Western blotting. (H to K) *ROCK1*, *PPARα*, *PGC1α*, and *ACOX1* mRNA levels. (L and M) Oil Red O staining (×200 magnification, bar = 200 μm/80 μm) and quantitative analysis of HK-2 cells with knockdown of the *ROCK1* gene. The data are presented as mean ± standard error of the mean (SEM) (*n* = 3/group). ^#^*P* < 0.05, ^##^*P* < 0.01, ^###^*P* < 0.001, and ^####^*P* < 0.0001, high-glucose (HG) vs. control; *P < 0.05, **P < 0.01, ***P < 0.001, and ****P < 0.0001, HG vs. ST; ns, not significant.

Building on this, we investigated the role of this pathway in HG-induced renal tubular injury in vitro. Results showed that HG stimulation notably up-regulated ROCK1 expression in HK-2 cells and increased the phosphorylation level of the p38 MAPK pathway (assessed by the P-p38/p38 ratio) while decreasing PPARα expression. ST intervention markedly reversed these HG-induced aberrations (Fig. 4A to C and G).

To further validate the regulatory role of ROCK1 in renal tubular lipid metabolism, we suppressed ROCK1 gene transcription in HK-2 cells using siRNA transfection. Following siROCK1 transfection, both the mRNA and protein expression levels of ROCK1 in HK-2 cells were reduced, with statistically significant differences confirmed by qPCR and WB (*P* < 0.05, Fig. 4D to F).

Subsequently, we examined changes in the expression of lipid-metabolism-related genes after HG treatment (Fig. 4H to K). Compared with the negative control transfection group (siCon group), the HG-treated negative control group (siCon-HG group) showed marked down-regulation of *PPARα*, *PGC1α*, and *ACOX1* mRNA expression. In contrast, the HG-treated ROCK1 knockdown group (siROCK1-HG group) exhibited noticeably higher mRNA expression of these key lipid metabolism genes than the siCon-HG group. Oil Red O staining further indicated that lipid deposition in the siROCK1-HG group was reduced compared with that in the siCon-HG group (Fig. 4L and M). The foregoing results confirm that *ROCK1* gene knockdown effectively alleviates HG-induced lipid dysregulation in renal tubular epithelial cells.

Collectively, these findings demonstrate that HG-induced up-regulation of ROCK1 promotes lipid accumulation in renal tubular epithelial cells, highlighting ROCK1 as a key regulator of renal lipid metabolism. Additionally, p38 MAPK phosphorylation-mediated inhibition of PPARα activity may represent a critical mechanism underlying lipid metabolism dysregulation. This confirms the potential of the p38 MAPK/PPARα axis as a therapeutic target for metabolic nephropathies and underscores that the homeostatic regulation of this axis is essential for maintaining renal metabolic balance.

### ST alleviated STZ-induced renal injury in DKD mice

**Fig. 5.**
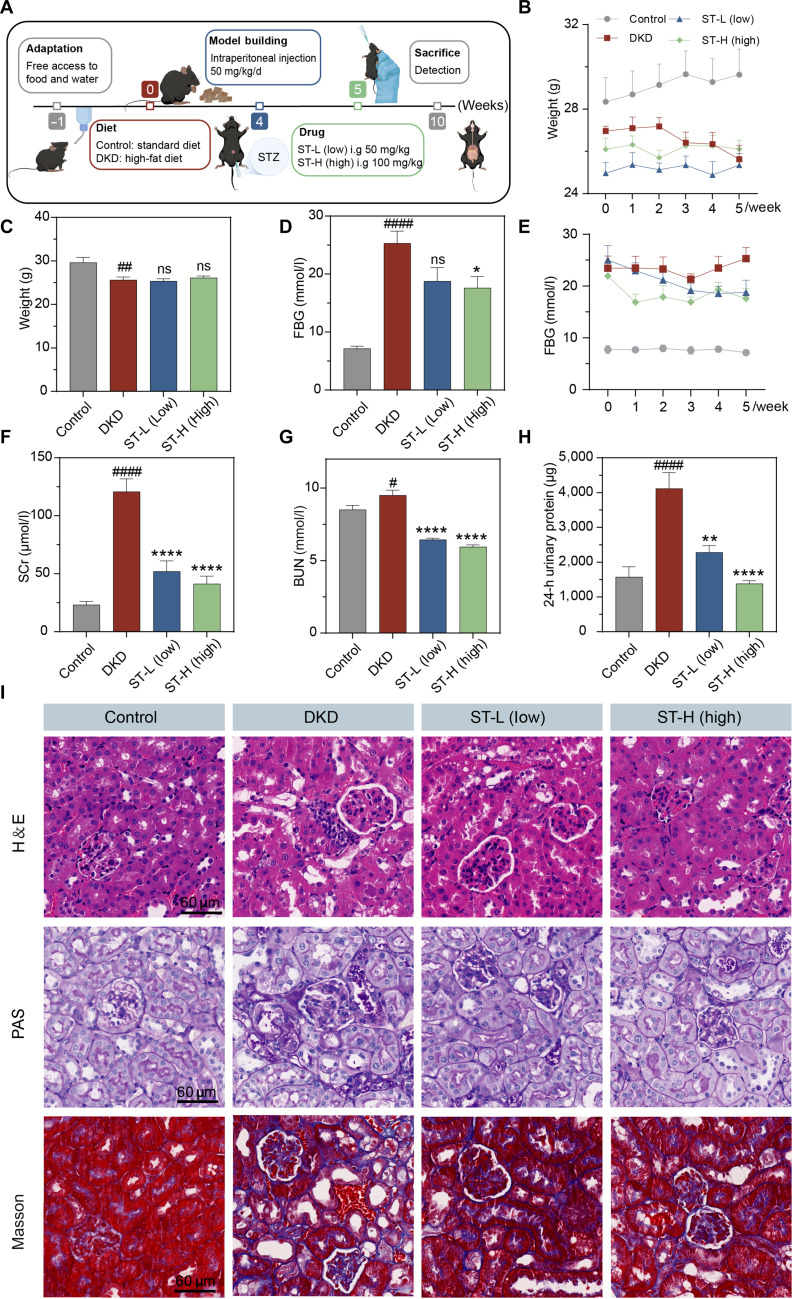
Stigmasterol (ST) alleviated streptozotocin (STZ)-induced renal injury in diabetic kidney disease (DKD) mice. (A) Schematic diagram of an animal experiment. (B) Body weight changes during treatment. (C) Final body weight. (D) Final fasting blood glucose levels. (E) Fasting blood glucose changes during treatment. (F) Serum creatinine levels. (G) Blood urea nitrogen levels. (H) 24-h urinary protein levels. (I) Renal histopathology (hematoxylin–eosin [H&E], periodic acid–Schiff [PAS], and Masson staining; ×200 magnification, scale bar = 60 μm). The data are presented as mean ± standard error of the mean (SEM) (*n* = 6/group). ^#^*P* < 0.05, ^##^*P* < 0.01, ^###^*P* < 0.001, and ^####^*P* < 0.0001, DKD vs. control; *P < 0.05, **P < 0.01, ***P < 0.001, and ****P < 0.0001, DKD vs. ST; ns, not significant.

As a phytosterol compound, ST has been shown to exhibit multiple biological activities: it not only ameliorates chronic inflammation associated with lipid metabolism disorders and regulates hepatic fatty acid metabolism but also reduces renal tissue lipid peroxidation levels and alleviates renal fibrosis in STZ-induced DKD rats. To validate the therapeutic effects of ST on DKD at the animal level, we established a DKD mouse model using STZ combined with an HFD. Mice were randomly assigned to ST low-dose (ST-L, 50 mg/kg) and ST high-dose (ST-H, 100 mg/kg) groups, with continuous intervention for 5 weeks. The experimental design is shown in Fig. 5A. During the intervention period, the FBG and body weight of mice were monitored weekly (Fig. 5B to E). Our results indicated that compared with the regular control group, DKD model mice exhibited significantly reduced body weight and markedly elevated FBG levels (*P* < 0.05), confirming the successful establishment of the DKD mouse model. In addition, the kidney weight and the ratio of kidney weight/body weight were up-regulated significantly in DKD model mice(*P* < 0.0001), and ST treatment could restore these changes (*P* < 0.01, Fig. S4). We further analyzed the glucose-lowering effect of ST: compared with the DKD model group, ST-L treatment reduced FBG levels, but the difference was not statistically significant (*P* > 0.05). In contrast, ST-H treatment significantly decreased FBG levels (*P* < 0.05). These findings indicate that ST exerts a dose-dependent glucose-lowering effect on DKD mice, with the ST-H group showing a more pronounced effect.

To evaluate the renoprotective effect of ST in DKD mice, we measured key renal function parameters, including SCr, BUN, urinary protein, and urine protein-to-creatinine ratio (UPCR). Compared with the normal control group, the DKD model group had significantly increased SCr, BUN, urinary protein, and UPCR levels (*P* < 0.05). After ST intervention, these abnormal renal function indicators were reduced to varying degrees (Fig. 5F to H and Fig. S5), demonstrating that ST effectively mitigates renal function impairment in DKD mice. A potential underlying mechanism may involve ST’s inhibition of renal oxidative stress and reduction of inflammatory injury—an observation consistent with reported preclinical findings in metabolic nephropathy research. To further assess the impact of ST on renal pathological morphology in DKD mice, we performed H&E, PAS, and Masson staining on renal tissue sections. DKD model mice displayed renal pathological damage, including glomerular basement membrane thickening (PAS staining), tubular vacuolar degeneration (H&E staining), abnormal renal glycogen deposition (PAS staining), and renal interstitial fibrosis (Masson staining). After intervention with different ST doses, these pathological changes were alleviated, with the ST-H group showing a more prominent improvement than the ST-L group (Fig. 5I). In summary, ST may alleviate DKD-related pathological damage by regulating renal fibrotic pathways, inhibiting inflammatory cascades, or promoting renal tissue repair—findings that further confirm its renoprotective effects.

### ST alleviated fibrosis and mitochondrial damage caused by lipid accumulation in DKD mice

**Fig. 6.**
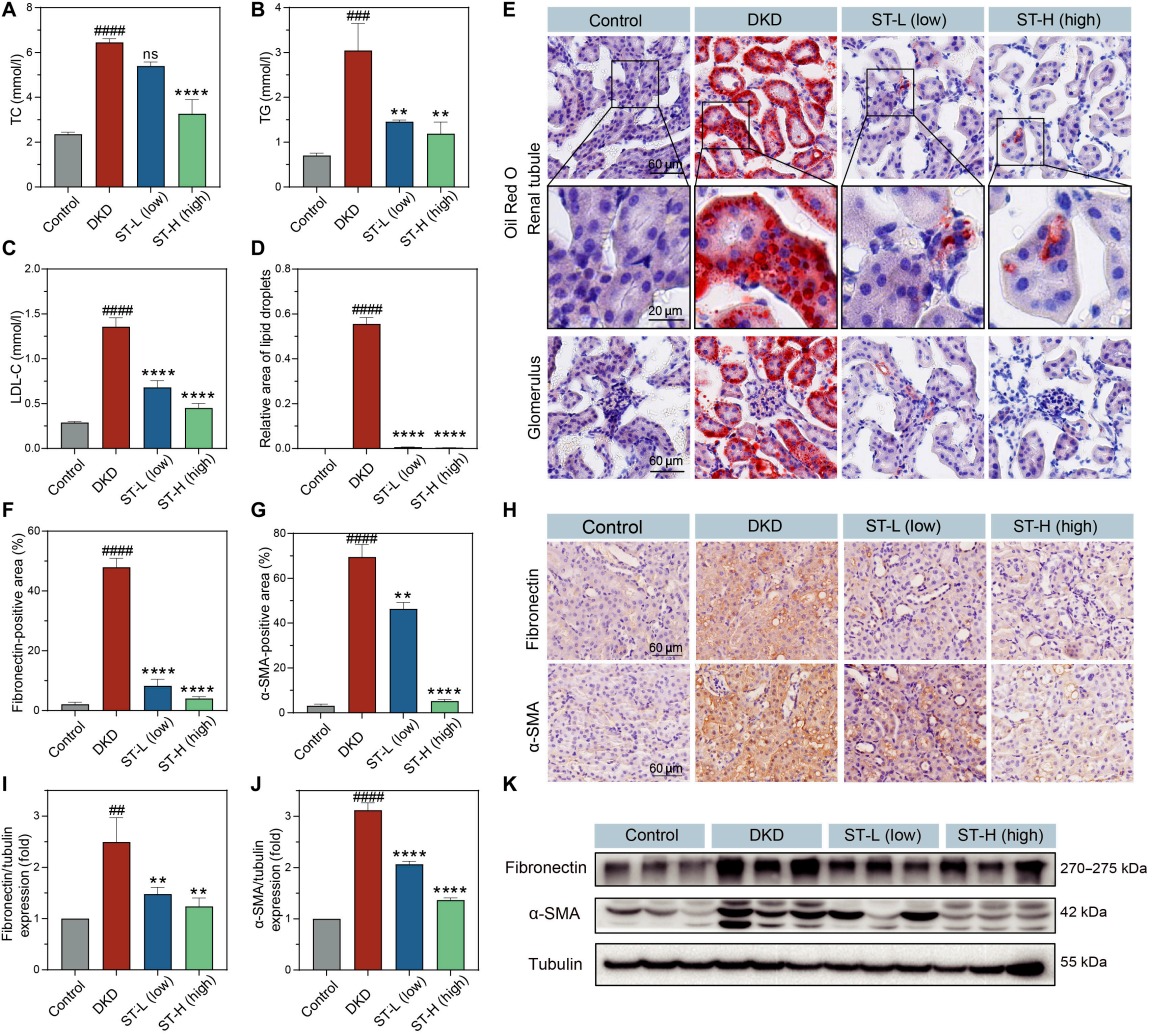
Stigmasterol (ST) alleviated fibrosis and mitochondrial damage caused by lipid accumulation in diabetic kidney disease (DKD) mice. (A) Total cholesterol levels in mouse serum. (B) Total triglyceride levels in mouse serum. (C) Serum low-density lipoprotein cholesterol levels in mice. (D) Quantitative map of Oil Red O in kidney tissue. (E) Oil Red O staining of mouse kidney tissue (glomeruli and tubules) (×200 magnification, bar = 60 μm/20 μm). (F and G) Immunohistochemical (IHC) staining of α-smooth muscle actin (α-SMA) and fibronectin for quantitative analysis. (H) IHC staining of α-SMA and fibronectin in mouse kidney tissue (×200 magnification, bar = 60 μm). (I and J) Quantitative analysis of α-SMA and fibronectin by protein blotting. (K) Protein blotting for the expression of α-SMA and fibronectin in renal tissues of each group. The data are presented as mean ± standard error of the mean (SEM) (*n* = 3/group). ^#^*P* < 0.05, ^##^*P* < 0.01, ^###^*P* < 0.001, and ^####^*P* < 0.0001, DKD vs. control; *P < 0.05, **P < 0.01, ***P < 0.001, and ****P < 0.0001, DKD vs. ST; ns, not significant.

TC, total TG, and LDL-C are core biomarkers for detecting lipid-metabolism-related disorders. In DKD, abnormalities in these 3 indicators serve as valuable references for disease monitoring. To this end, we performed serological analyses. Results showed that serum TC, TG, and LDL-C levels were significantly elevated in DKD mice (*P* < 0.001), indicating the presence of dyslipidemia during DKD progression (Fig. 6A to C). Consistently, the results of TG and TC assays in renal tissues showed an identical trend (Fig. S6): TG and TC levels were markedly elevated in DKD model mice, whereas both low- and high-dose ST interventions lead to a significant reduction in TG and TC concentrations (*P* < 0.0001). To further characterize renal lipid metabolism abnormalities in DKD mice, we assessed the extent of renal lipid accumulation via Oil Red O staining of mouse kidney frozen sections. The DKD group exhibited a marked increase in Oil Red O-positive staining areas (indicative of lipid deposition) in renal tissues, confirming severe renal lipid accumulation. Comparative analysis revealed that lipid accumulation was more pronounced in the renal tubular region than in the glomeruli. Notably, intervention with different doses of ST markedly alleviated this renal lipid accumulation in DKD mice (Fig. 6D and E). It is well established that hypercholesterolemia and hypertriglyceridemia promote abnormal lipid deposition in renal tissues, which in turn induces renal structural damage and functional decline. Concurrently, renal tissue lipid overload inhibits FAO and directly triggers mitochondrial dysfunction—manifested by increased reactive ROS production and mitochondrial swelling/deformation. Mitochondrial damage further exacerbates energy metabolism imbalance in renal cells, ultimately accelerating renal fibrosis through multiple pathways. Based on these pathological mechanisms, we subsequently measured the expression levels of key fibrosis-related factors in the mouse renal cortex using IHC and WB (Fig. 6F to K). Compared with the normal control group, DKD model mice showed notable up-regulation of fibronectin and α-SMA. After ST intervention, the expression of these 2 fibrosis markers was reduced, suggesting that ST can inhibit renal fibrosis progression in DKD mice by regulating relevant signaling pathways.

**Fig. 7.**
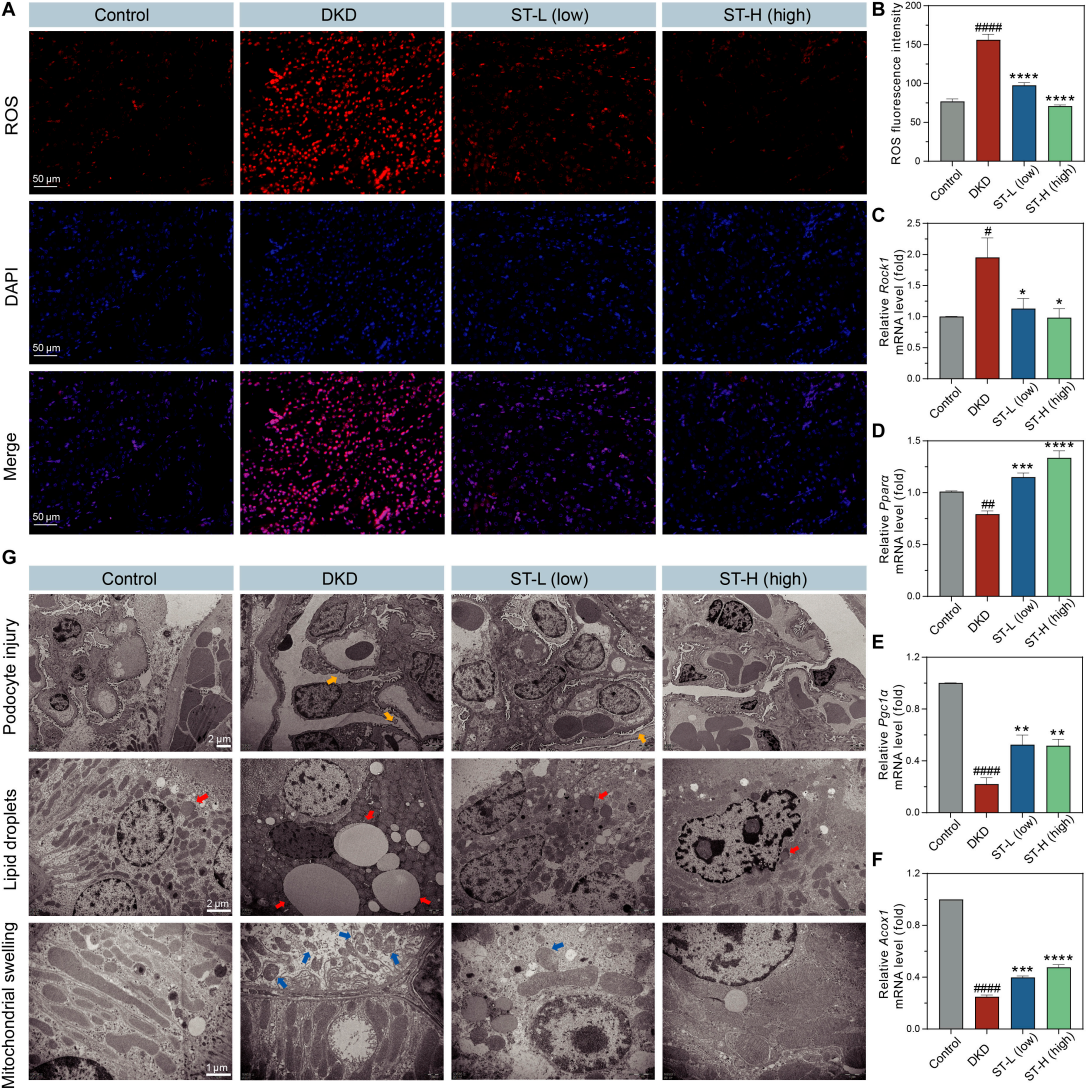
Stigmasterol (ST) reduces mitochondrial dysfunction in diabetic kidney disease (DKD) mice. (A) Reactive oxygen species (ROS) staining of mouse kidney tissue (bar = 50 μm). (B) Quantification of ROS fluorescence intensity. (C to F) Messenger RNA (mRNA) levels of *Rock1*, *Pparα*, *Pgc1α*, and *Acox1* in mouse kidney. (G) Effects of ST on ultrastructure in the kidneys of DKD mice (yellow arrows indicate podocyte injury, red arrows indicate lipid droplet accumulation, and blue arrows indicate mitochondrial swelling). The data are presented as mean ± standard error of the mean (SEM) (*n* = 3/group). ^#^*P* < 0.05, ^##^*P* < 0.01, ^###^*P* < 0.001, and ^####^*P* < 0.0001, DKD vs. control; *P < 0.05, **P < 0.01, ***P < 0.001, and ****P < 0.0001, DKD vs. ST; ns, not significant.

To explore the molecular mechanism by which ST regulates renal lipid metabolism in DKD mice, we examined the mRNA expression levels of genes involved in lipid metabolism and FAO. Results showed that compared with that of the normal control group, the mRNA expression of *Pparα*, *Pgc1α*, and *Acox1* was considerably down-regulated in the kidneys of DKD model mice (Fig. 7C to F). This finding indicates that renal FAO is markedly inhibited in DKD, and such impairment of FAO is often associated with mitochondrial dysfunction. In contrast, ST intervention up-regulated the mRNA expression of these lipid-metabolism-related genes, demonstrating that ST can promote renal fatty acid β-oxidation by enhancing the expression of key metabolic genes, thereby alleviating abnormal lipid accumulation. Excessive reactive ROS production is a key hallmark of mitochondrial dysfunction. Our data revealed that renal ROS levels were markedly higher in the DKD model group than in the normal control group. After ST treatment, renal ROS levels in DKD mice were markedly suppressed (Fig. 7A and B), further confirming the protective effect of ST on renal mitochondrial function in DKD mice. To directly visualize renal structural pathological changes in DKD mice and the interventional effect of ST, we analyzed renal tissue ultrastructure using transmission electron microscopy (Fig. 7G). Data analysis showed the following: Kidneys from the normal control group had mitochondria with regular morphology and intact structure, and kidneys from the DKD model group exhibited mitochondrial pathological damage, characterized by mitochondrial inner chamber swelling, crista shortening, and a reduction in number, and even rupture of the inner mitochondrial membrane in some cases. Concurrently, glomerular podocyte foot process fusion and loss were observed, along with an increased number and size of lipid droplets in the renal tubular lumen; ST intervention mitigated these ultrastructural pathological changes in the kidney. The renal tubule is the core site for transmembrane solute transport, and its physiological functions rely on a substantial energy supply. Fatty acids serve as the primary energy substrate for renal tissue, and fatty acid β-oxidation is the preferred pathway for renal tubular epithelial cells (especially proximal tubular cells) to generate adenosine triphosphate (ATP), which is essential to meet the high energy demands of ion transport. Since fatty acid β-oxidation primarily occurs in mitochondria, proximal tubular cells are densely populated with mitochondria to ensure high levels of ATP production. Impairment of mitochondrial fatty acid β-oxidation directly leads to insufficient ATP synthesis and accumulation of lipotoxicity, ultimately exacerbating renal structural damage and functional decline.

### ST inhibits ROCK1/p38 MAPK/PPARα signaling pathway in DKD mice

**Fig. 8.**
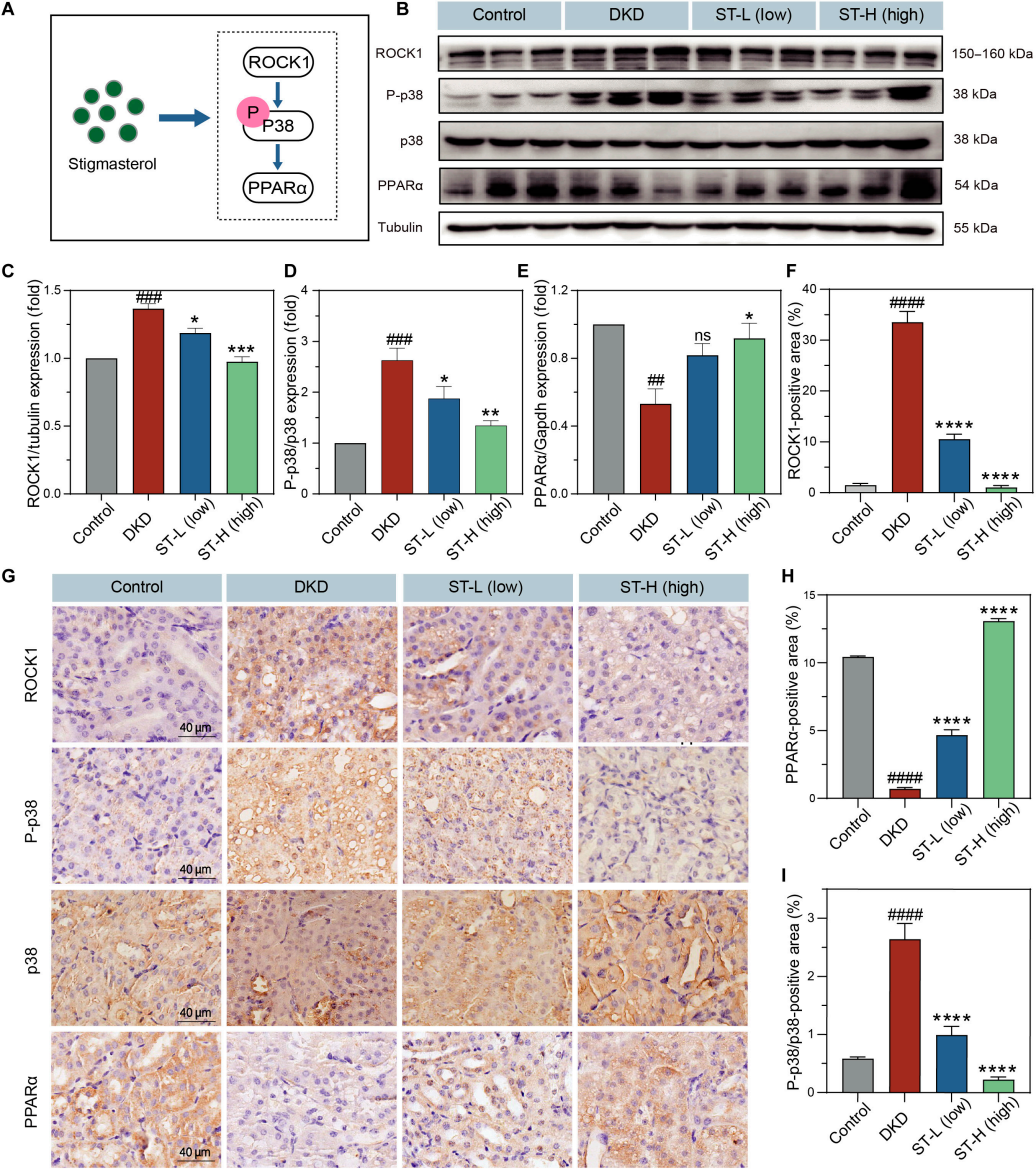
Stigmasterol inhibits the rho-associated coiled-coil protein kinase 1 (ROCK1)/p38 mitogen-activated protein kinase (MAPK)/peroxisome proliferator-activated receptor α (PPARα) signaling pathway in diabetic kidney disease (DKD) mice. (A) The mechanism of DKD mice. (B) Protein blotting assay for the expression of ROCK1, P-p38, p38, and PPARα in renal tissues. (C to E) Quantitative analysis of ROCK1, P-p38/p38, and PPARα by protein blotting. (F, H, and I) Quantitative analysis of immunohistochemical (IHC) staining for ROCK1, P-p38/p38, and PPARα. (G) IHC staining of mouse kidney tissues for ROCK1, P-p38, p38, and PPARα (×200 magnification, bar = 40 μm). The data are presented as mean ± standard error of the mean (SEM) (*n* = 3/group). ^##^*P* < 0.01, ^###^*P* < 0.001, and ^####^*P* < 0.0001, DKD vs. control; *P < 0.05, **P < 0.01, ***P < 0.001, and ****P < 0.0001, DKD vs. ST; ns, not significant.

To clarify the regulatory effect of ST on key signaling pathways in the kidneys of DKD mice, we measured the protein expression levels of ROCK1, p38 MAPK (including its phosphorylated form, P-p38), and PPARα in renal tissues via WB (Fig. 8A). Results showed the following: (a) ROCK1 protein expression in the DKD model group was significantly higher than that in the normal control group (*P* < 0.05). ST intervention significantly reduced ROCK1 protein expression, with a more pronounced decrease in the ST-H group (*P* < 0.05). (b) The expression level of P-p38 protein in the DKD model group was significantly increased (*P* < 0.05), while no significant difference in total p38 protein expression was observed among all groups (*P* > 0.05). Consequently, the P-p38/p38 ratio in the model group was significantly higher than that in the normal control group (*P* < 0.05). After ST treatment, this ratio decreased in a dose-dependent manner, with the ST-H group showing a more significant effect (*P* < 0.05). (c) PPARα protein expression in the DKD model group was significantly lower than that in the normal control group (*P* < 0.05). ST intervention markedly up-regulated PPARα protein expression, and the increase in the ST-H group was more prominent (Fig. 8B to E). IHC results further validated the above WB findings: compared with the normal control group, the DKD model group exhibited a significantly increased ROCK1-positive expression area, a notably higher P-p38/p38 positive signal ratio, and a markedly reduced PPARα-positive expression area (all *P* < 0.05). After ST intervention, ROCK1-positive expression in renal tissues was strongly decreased, the P-p38/p38 positive signal ratio was notably reduced, and PPARα-positive expression was restored (Fig. 8F to I). The regulatory effect was more pronounced in the ST-H group than in the ST-L group, indicating that ST can modulate the abnormal activation of the ROCK1/p38 MAPK/PPARα axis at the tissue level.

## Conclusion

DKD is a major microvascular complication of diabetes mellitus. Its pathological progression is accompanied by multidimensional dysregulation of the renal metabolic network and ultimately leads to renal fibrosis, making it a primary cause of ESRD. Currently, clinical practice has established multitarget therapeutic strategies, including blood glucose control, blood pressure regulation, and administration of renoprotective agents. While these approaches can delay DKD progression to a certain extent, they are constrained by the complexity of its pathological mechanisms, and completely halting DKD progression remains a major challenge.

ROCK1 is a key member of the serine/threonine kinase family. It regulates cytoskeletal rearrangement to participate in core biological processes. A study has confirmed that ROCK1 activation promotes mesangial cell proliferation in DKD, accelerates glomerulosclerosis, and drives renal inflammation and fibrosis by modulating the expression of inflammatory signaling pathways and fibrotic factors [13]. However, it has not established a direct molecular link between ROCK1 and 2 critical pathological features of DKD: “renal lipid metabolism disorder” and “mitochondrial damage”. To investigate the specific relationship between the 2, we performed a series of studies. The results confirmed that the abnormal activation of ROCK1 is a “molecular switch” in the vicious cycle of “lipid deposition → mitochondrial dysfunction → oxidative stress → fibrosis” in DKD, and ST directly binds ROCK1 through high affinity (rather than indirect regulation), blocking the switch from the source. This mechanism fills the research gap of “natural molecule targeting ROCK1 to improve renal metabolic damage” and gives “metabolic regulation new attributes” to the role of ROCK1 in metabolism-related nephropathy.

The core difficulty of DKD renal fibrosis lies in the superposition of multiple pathological links (lipid toxicity, mitochondrial damage, inflammation, and fibrosis promote each other), and the effect of single mechanism intervention is limited. Based on this, this study confirmed that ST can simultaneously achieve the following by inhibiting ROCK1: (a) down-regulation of p38 MAPK/up-regulation of PPARα to improve lipid metabolism (solve the problem of “lipid accumulation”) and (b) relieving of mitochondrial membrane swelling and reduction of ROS (solve the problem of “energy metabolism disorder and oxidative damage”). This “dual-pathway synergy” regulatory model has for the first time clarified that natural molecules can interfere with the “multicore pathological links” of DKD through a single target (ROCK1), providing a clear logical chain of “target–pathway–effect” for understanding the “pleiotropic effects of natural products”, rather than the “pan-effect regulation” in traditional cognition. The importance of this discovery is not limited to DKD only, but it also has extensive enlightening value for “research on metabolism-related nephropathy” and “mechanism mining of natural products”.

This study still has several limitations: First, the specific spatial structure and interaction sites between ST and ROCK1 have not been clarified and require further investigation. Second, although this study confirms that ST can target and bind to ROCK1 and down-regulate its expression, the exact regulatory mechanism and whether it is related to ubiquitination need further verification. Third, the effect of ST on alleviating DKD by reducing lipid deposition has been demonstrated only in animal models or cell-based experiments, and clinical trial validation is still lacking.

## Data Availability

All data generated or analyzed during this study are included in this published article and its supplementary materials files.
