# Supplementary material for: Stigmasterol-Mediated Targeting of Rho-Associated Coiled-Coil Protein Kinase 1 Ameliorates Diabetic Kidney Disease and Attenuates Renal Tubular Lipid Deposition
Source: Biomater Res. 2026 Jul 17;30:0389. doi: 10.34133/bmr.0389 (PMC13376380; doi:10.34133/bmr.0389)
Supplement: Supplementary 1 — Supplementary Materials and Methods Figs. S1 to S6 Tables S1 to S4 [file bmr.0389.f1.docx]

**SUPLEMENTAL MATERIALS**

**Stigmasterol-Mediated Targeting of Rho-Associated Coiled-Coil Protein Kinase 1 Ameliorates Diabetic Kidney Disease and Attenuating Renal Tubular Lipid Deposition**

Yuchi Chen,a,1 Xinyao Xu,a,1 Ningning Yuan,a,d,1 Yangtian Yan,a Yuxin Ye,a Zhuoen He,a Jinyue He,a Chi Zhang,a Hao Wang,a Haitao Yuan,b,e∗ Jianxin Diao,a,∗ Wei Xiao,a,c,e∗

^a^School of Traditional Chinese Medicine, Southern Medical University, Guangzhou, Guangdong, 510515, China

^b^Center for Drug Research and Development, Guangdong Provincial Key Laboratory of Pharmaceutical Preparations Research and Evaluation, Guangdong Pharmaceutical University, Guangzhou,510006, China

^c^Guangdong Provincial Key Laboratory of Autophagy and Major Chronic Non-communicable Diseases, Affiliated Hospital of Guangdong Medical University, Zhanjiang, Guangdong 524001, China

^d^College of Life Science, Zhuhai College of Science and Technology, Zhuhai, 519090, China

^e^Key Laboratory of Glucolipid Metabolic Disorder, Ministry of Education, Guangdong Pharmaceutical University, Guangzhou, Guangdong, 510006, China

*Corresponding author:

Wei Xiao

E-mail: [xw7688@smu.edu.cn](mailto:xw7688@smu.edu.cn)

Jianxin Diao

E-mail: [jianxindiao@163.com](mailto:jianxindiao@163.com)

Haitao Yuan

E-mail: yht193525@163.com

# Supplementary Materials and Methods

**1. Quantitative PCR**

Total RNA was extracted using TRIzol (R1100, Solarbio, Beijing, China) and reverse-transcribed using HiScript II Q RT SuperMix (R223, Vazyme, Nanjing, China). QPCR was performed on a LightCycler 96 system with primers. Data were analyzed by the 2^−ΔΔCt^ method.

**2. Molecular docking**

The two-dimensional structure of stigmasterol was downloaded from the PubChem database and converted into a three-dimensional (3D) structure using Chem3D 22.0.0, which was then transformed into the PDBQT format. Retrieval of the ROCK1 protein crystal structure (PDB ID: 2ETR) was performed from the PDB database; subsequent removal of water molecules and addition of hydrogen atoms to the protein were carried out using PyMOL to maintain a proper protonation state. The processed stigmasterol and ROCK1 protein were subjected to docking simulation via AutoDock Vina. The docking results were subsequently visualized using PyMOL, with interactions analyzed based on hydrogen bonds, hydrophobic effects, and other forces. The optimal conformation was screened according to binding affinity and interaction mode.

**3. Cellular thermal shift assay (CETSA)**

Cells were seeded into 10-cm culture dishes at 70% confluency. After seeding, the cells were treated with 15 μM stigmasterol or an equal volume of DMSO and incubated for 24 h. Following three washes with PBS, cells were scraped from the dishes using 500 μL of PBS. The cell suspension was then aliquoted into 13 tubes at 40 μL per tube, labeled with temperature points ranging from 54°C to 78°C (2°C gradient interval). Each sample was incubated in a metal bath at the designated temperature for 3 min, followed by immediate termination of the reaction via ice bath. Protease and phosphatase inhibitors were added to the samples, which were then snap-frozen in liquid nitrogen and subjected to three cycles of thawing at 37°C with shaking between cycles. Following centrifugation at 14,000 × g for 15 min, 32 μL of supernatant was combined with 8 μL of 5× loading buffer; the mixture was then heat-denatured at 95 °C for 5 min using a metal bath. Finally, target protein expression was detected by Western blot analysis.

**4. Surface plasmon resonance (SPR)**

First, chip activation was performed: 0.765 g of EDC and 0.115 g of NHS were separately dissolved in 10 mL of H₂O, mixed, and used to immerse 3D Dextran chips. The chips were incubated on a shaker for 15–20 min. After activation, the chips were briefly rinsed, air-dried, and spotted with ROCK1 active protein on their surfaces using a bio-dotting instrument. Following spotting, the chips were sealed, labeled, and incubated at 4°C for ≥1 h. Stigmasterol was gradient-diluted in 0.1% DMSO-PBS solution (0–100 μM). For SPR detection, different concentrations of stigmasterol solutions were sequentially injected (flow rate: 20–50 μL/min, 27°C), and changes in response units (RU) were recorded with three replicates per concentration. Data were baseline-corrected, and solvent effects were subtracted using BIA evaluation software. Binding curves were fitted to a 1:1 Langmuir model to calculate the dissociation constant, and binding graphs were generated using OriginLab.

**5. ROS measurement**

(1) Using a microtome, gently transfer 5 μm-thick sections of renal cortical tissue onto a microscope slide and allow them to air-dry at room temperature for 15 minutes;

(2) Take the DHE probe stock solution (concentration: 10 mM) (DHE, S0063, Beyotime, Shanghai, China) and dilute it with PBS at a ratio of 1:1000 to prepare the DHE working solution (final concentration: 10 μM). Gently invert to mix, ensuring all steps are performed in the dark (to prevent light exposure from causing probe degradation); place the dried tissue sections in PBS and rinse three times at room temperature for 5 minutes each time to remove impurities and residual OCT embedding medium from the surface of the sections; Blot excess moisture from the edges of the slides with filter paper; use a histochemical pen to mark a circle on the tissue section; apply the DHE working solution in uniform droplets, ensuring complete coverage of all tissue areas; incubate at 37°C in the dark for 30 minutes;

(3) Upon completion of the incubation, gently decant the DHE working solution. Place the slides in PBS and rinse three times at room temperature, protected from light, for 5 minutes each time. Subsequently, add DAPI staining solution and incubate at room temperature, protected from light, for 10 minutes. After decanting, place the slides in PBS and rinse three times at room temperature, protected from light, for 5 minutes each time, to thoroughly remove unbound stain and avoid fluorescent background interference.

(4) Remove the rinsed slides, blot the edges dry with filter paper, and apply 1–2 drops of anti-fade mounting medium to the centre of the tissue section. Gently place a coverslip over the sample using forceps, taking care to avoid creating air bubbles (as these may affect fluorescence observation); Perform fluorescence imaging using a Nikon Eclipse C1 microscope equipped with the appropriate excitation/emission filters (518 nm excitation/585 nm emission).

(5) Perform a semi-quantitative analysis of ROS production by measuring the fluorescence intensity of oxidised DHE (red fluorescence) using ImageJ software (NIH).

**6. ROCK1 ELISA**

The concentration of ROCK1 in HK-2 cell lysates was measured using a sandwich enzyme-linked immunosorbent assay (ELISA). After washing the cells with PBS, they were lysed by sonication and centrifuged to collect the supernatant. Standards (0.63–40 ng/mL) and samples were equilibrated to room temperature prior to analysis. Microplates pre-coated with anti-ROCK1 capture antibody were loaded with 100 μL of standards or samples per well and incubated at 37°C for 2 h. Following washing steps, biotinylated detection antibody (1:100 dilution, 37°C for 1 h), streptavidin-HRP conjugate (1:100 dilution, 37°C for 1 h), and TMB substrate (incubated in the dark for 15–20 min) were sequentially added. The reaction was terminated with 50 μL stop solution. Absorbance was measured at 450 nm (with 630 nm as a reference wavelength), and ROCK1 concentrations were calculated using a four-parameter logistic (4-PL) regression model based on the standard curve.

*
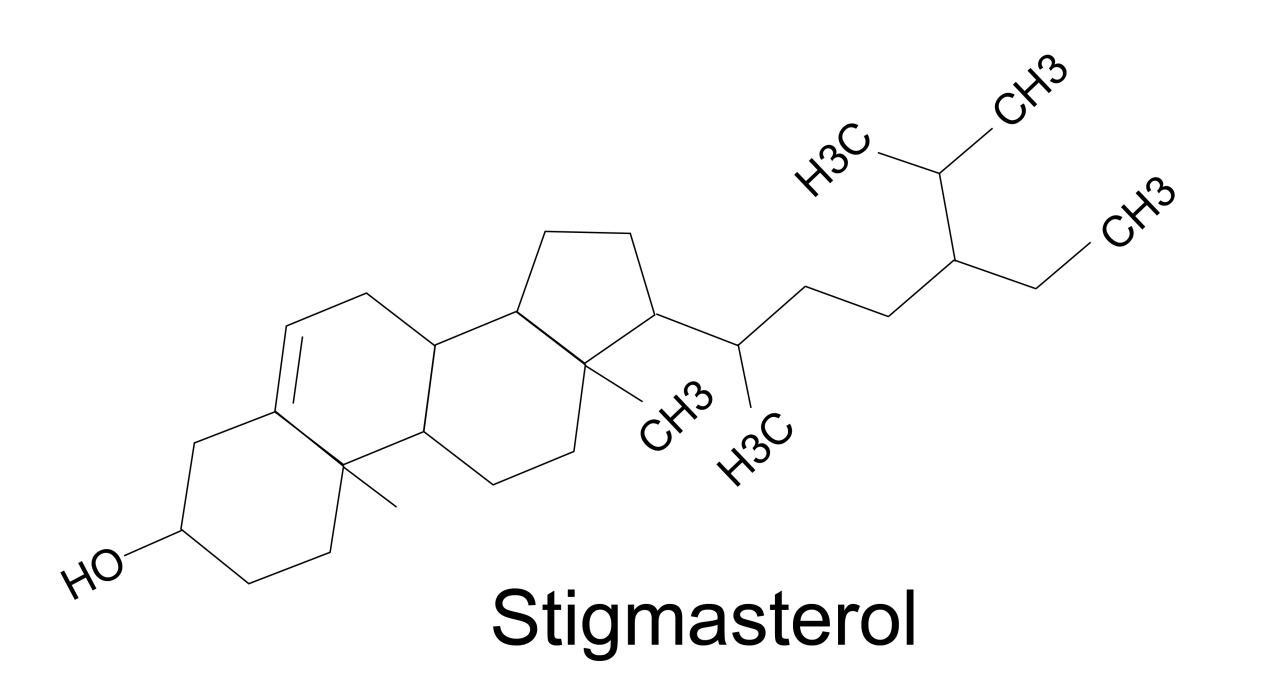
*

**Figure S1. Molecular structure of ST.**


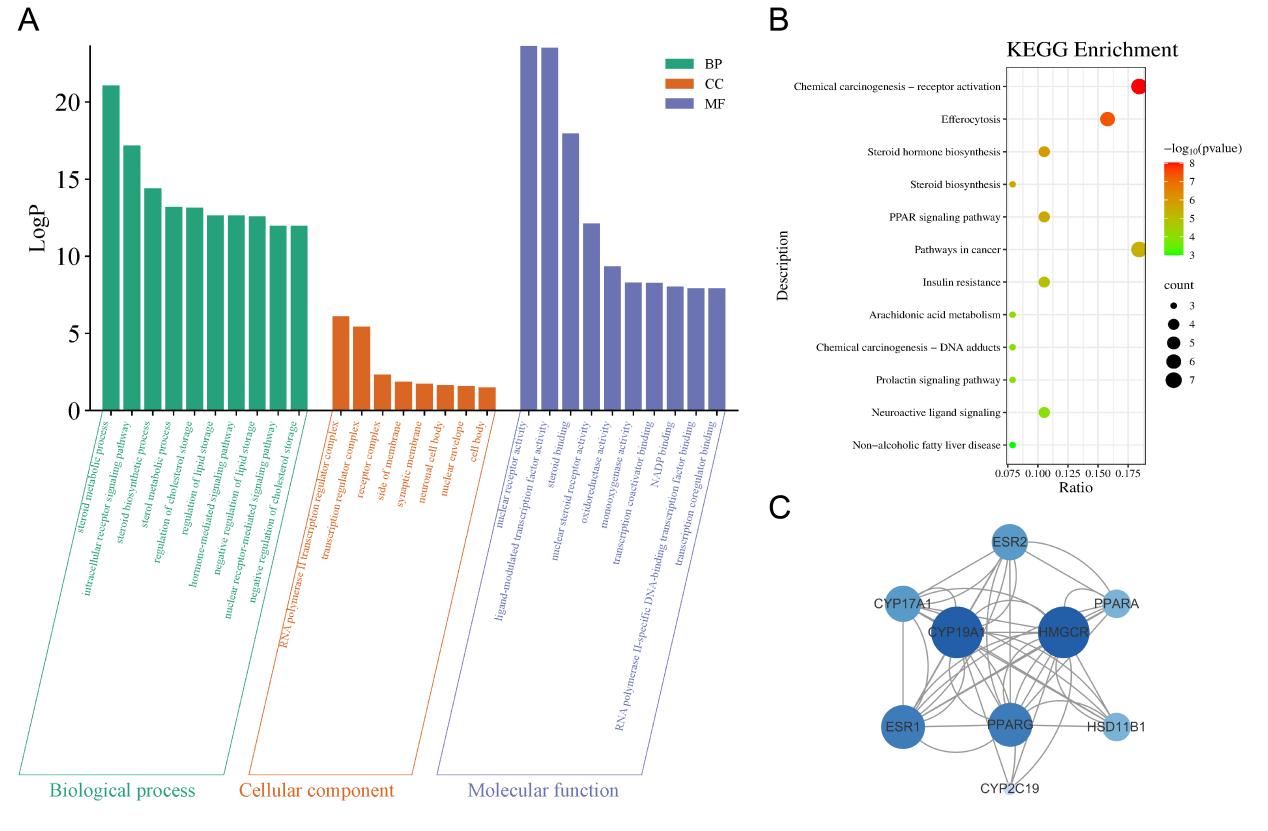


**Figure S2. Effect of ST on DKD via network pharmacology.**

(A) GO enrichment analysis results of ST in DKD, visualized as a histogram. BP: dark cyan, CC: sienna, and MF: steel blue. (B) KEGG pathway enrichment analysis results visualized as a bubble plot. X-axis: gene ratio; Y-axis: enriched pathways; bubble color: p-value threshold; bubble size: number of related genes.(C)Nodes represent ST/DKD target genes; edges indicate potential molecular interactions between their encoded proteins. Node size/color reflects network centrality (larger nodes = key hub molecules). This diagram visualizes the intricate regulatory links among these targets.


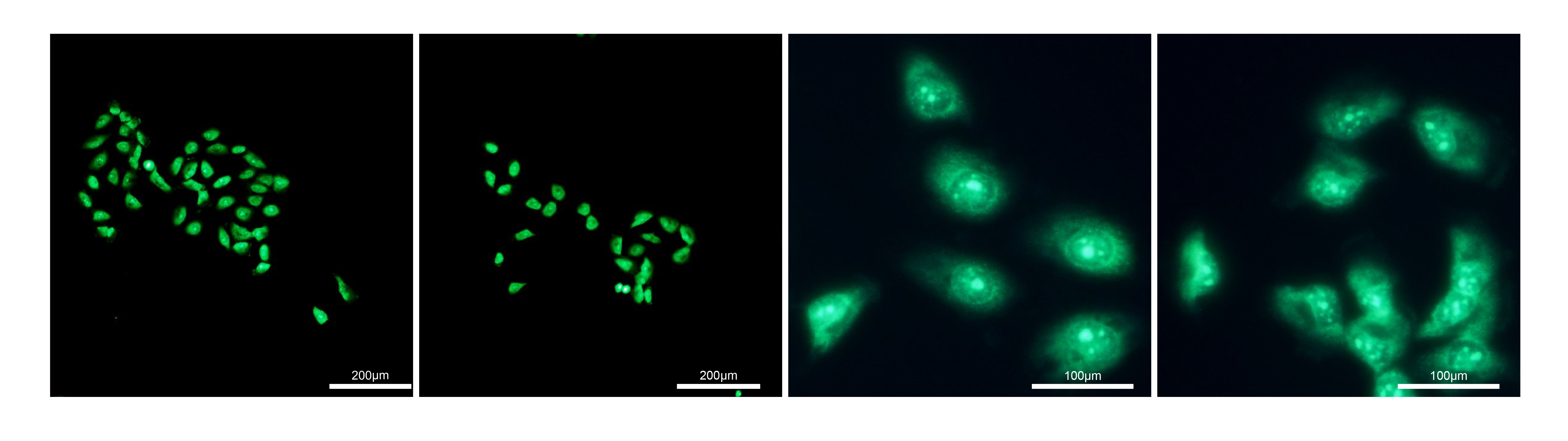


**Figure S3. Mycoplasma detection result of HK-2 cells (negative). scale bar = 100/ 200 μm.**


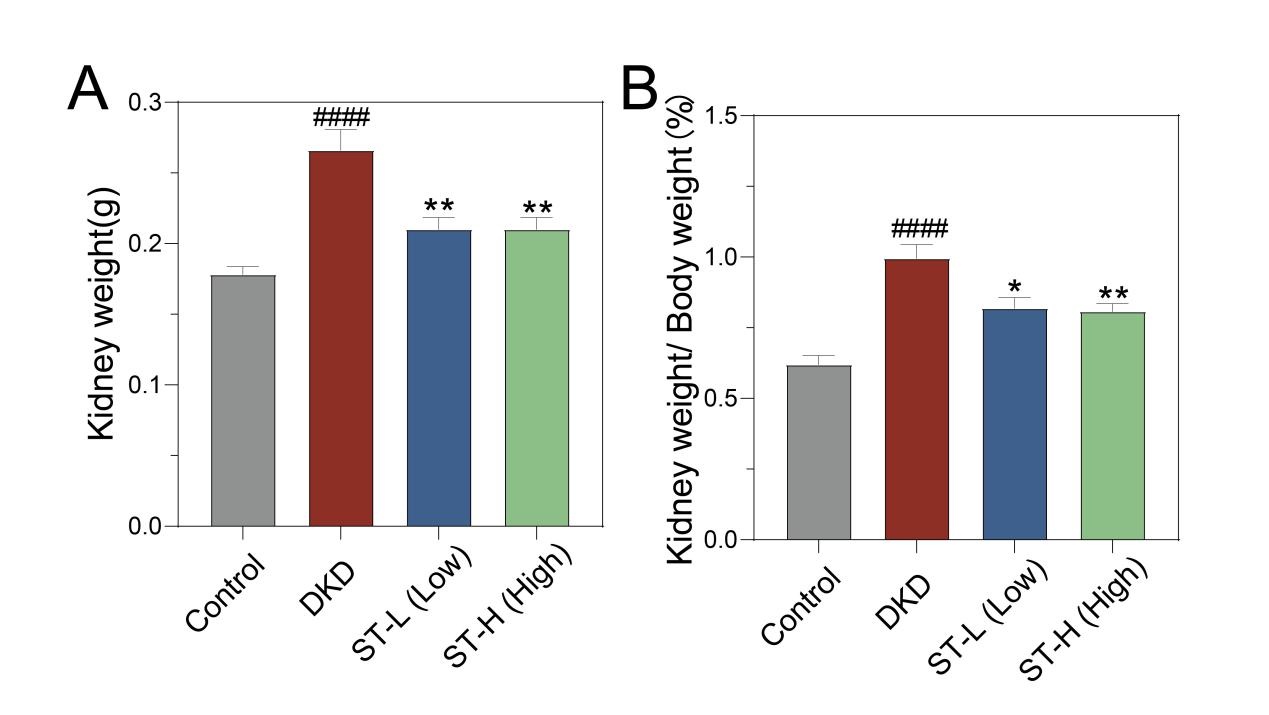


**Figure S4. Kidney weight and Kidney weight/ Body weight.**

1. Kidney weight (n=5). (B) Kidney weight/ Body weight (n=5). The data are presented as the mean ± SEM. ^####^*p* < 0.0001 DKD vs. Control; **p* < 0.05; ***p* < 0.01; *****p* < 0.0001 DKD vs. ST.

**Figure S5. Effects of ST on Urine Protein-to-Creatinine Ratio (UPCR) in DKD mice.**

The UPCR levels were measured in the Control, DKD, and ST-treated (Low, High dose) at week 5. Data are presented as mean ± SEM (n = 3 per group). ^#^*p* < 0.05 DKD vs. Control; **p* < 0.05 ST vs. DKD.


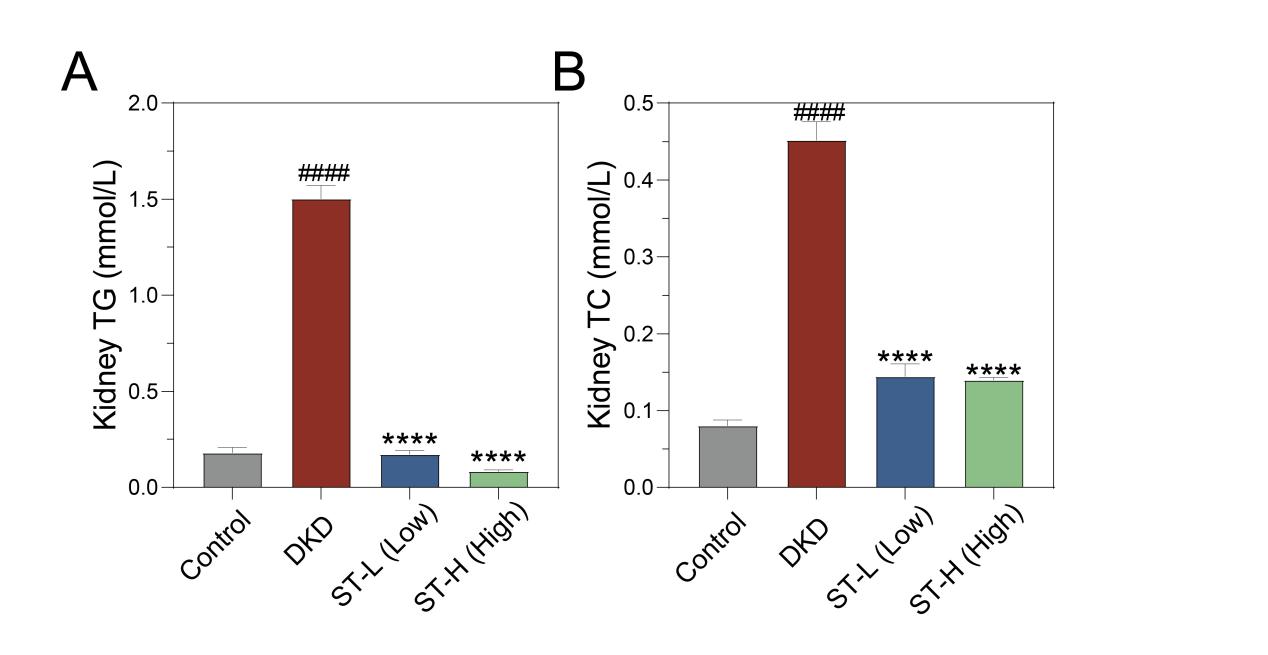


**Figure S6. Kidney TG and TC content.**

(A) Total triglyceride levels in mouse kidney (n=3). (B) Total cholesterol levels in mouse kidney. (n=3).The data are presented as the mean ± SEM. ^####^*p* < 0.0001 DKD vs. Control; **p* < 0.05; ***p* < 0.01; *****p* < 0.0001 DKD vs. ST.

**Table S1. Diabetic kidney disease samples**

| Sample Name | Log2 GFR (MDRD) (ml/min/1.73m2) | Median-centered Log2 ROCK1 (reporter ENSG00000067900) Expression Value |
| --- | --- | --- |
| 28305 | 5.3928266 | 5.65975 |
| 28307 | 5.7517176 | 5.63977 |
| 28308 | 3.4544222 | 6.06826 |
| 28310 | 3.3707268 | 6.21671 |
| 28312 | 4.376542 | 5.98081 |
| 28313 | 6.1829715 | 5.77844 |
| 28316 | 6.0149913 | 5.47636 |
| 28318 | 5.6059732 | 6.00381 |

**Table S2. siRNA primer sequences**

| Gene | Primer |
| --- | --- |
| H6093-siROCK1-2 sense | GGUUGGAACUUACAGUAAA(dT)(dT) |
| H6093-siROCK1-2 antisense | UUUACUGUAAGUUCCAACC(dT)(dT) |
| NC sense | AAAA |
| NC antisense | UUUU |

**Table S3. qPCR primer sequences.**

| Species | Gene names | Primer |
| --- | --- | --- |
| Human | CYPD-F | CGCTTTCCTGACGAGAACTTT |
| Human | CYPD-R | TCTTTGACGTGACCGAACACA |
| Human | ANT1-F | CTCTCCTTCTGGAGGGGTAAC |
| Human | ANT1-R | GAACTGCTTATGCCGATCCAC |
| Human | VDAC1-F | CTGACCTTCGATTCATCCTTCTC |
| Human | VDAC1-R | CTCCCGCTTGTACCCTGTC |
| Human | VDAC2-F | GGCGTGGAATTTTCAACGTCC |
| Human | VDAC2-R | AGACCATACTCACACCACTTGTA |
| Human | PPARα-F | TTTCCGCTTGACCCTTCCTC |
| Human | PPARα-R | GAAACGTATCAGCGCAGCAG |
| Human | GAPDH-F | GTCTCCTCTGACTTCAACAGCG |
| Human | GAPDH-R | ACCACCCTGTTGCTGTAGCCAA |
| Human | PGC1α-F | CACCAGCCAACACTCAGCTAAG |
| Human | PGC1α-R | AGGGTCATCGTTTGTGGTCAG |
| Human | ACOX1-F | CCCGAAAGCCTAACCGAAGCATA |
| Human | ACOX1-R | CATCATAGCGGCCAAGCACAGAG |
| Human | ROCK1-F | GAAACAGTGTTCCATGCTAGACG |
| Human | ROCK1-R | GCCGCTTATTTGATTCCTGCTCC |
| Mouse | Pgc1α-F | TATGGAGTGACATAGAGTGTGC |
| Mouse | Pgc1α-R | CCACTTCAATCCACCCAGAAAG |
| Mouse | Pparα-F | AGAGCCCCATCTGTCCTCTC |
| Mouse | Pparα-R | ACTGGTAGTCTGCAAAACCAAA |
| Mouse | Acox1-F | TAACTTCCTCACTCGAAGCCA |
| Mouse | Acox1-R | AGTTCCATGACCCATCTCTGTC |
| Mouse | Gapdh-F | AGGTCGGTGTGAACGGATTTG |
| Mouse | Gapdh-R | TGTAGACCATGTAGTTGAGGTCA |
| Mouse | Rock1-F | CACGCCTAACTGACAAGCACCA |
| Mouse | Rock1-R | CAGGTCAACATCTAGCATGGAAC |

**Table S4. Binding affinity of molecular docking for ROCK1**

| Number | Monomer | Pubchem Number | Molecular Formula | Vina |
| --- | --- | --- | --- | --- |
| 1 | Stigmasterol | 5280794 | C29H48O | -8.8 |
| 2 | Cyclopamine | 442972 | C27H41NO2 | -8.8 |
| 3 | Oleanolic acid | 10494 | C30H48O3 | -8.7 |
| 4 | Formononetin | 5280378 | C16H12O4 | -8.2 |
| 5 | Glycitin | 187808 | C22H22O10 | -8.1 |
| 6 | Astragaloside IV | 13943297 | C41H68O14 | -8.1 |
| 7 | Astragaloside II | 13996693 | C43H70O15 | -8.1 |
| 8 | Methylnissolin-3-O-glucoside | 101679160 | C23H26O10 | -8 |
| 9 | Calcifediol | 5283731 | C27H44O2 | -7.9 |
| 10 | Calycosin | 5280448 | C16H12O5 | -7.8 |
| 11 | Luteolin | 5280445 | C15H10O6 | -7.8 |
| 12 | Medicarpin | 336327 | C16H14O4 | -7.7 |
| 13 | Vitamin D3 | 5280795 | C27H44O | -7.7 |
| 14 | Epicatechin | 72276 | C15H14O6 | -7.6 |
| 15 | Chryseriol | 5280666 | C16H12O6 | -7.6 |
| 16 | Ellagic acid | 5281855 | C14H6O8 | -7.5 |
| 17 | Ononin | 442813 | C22H22O9 | -7.5 |
| 18 | Quercetin | 5280343 | C15H10O7 | -7.4 |
| 19 | Kaempferol | 5280863 | C15H10O6 | -7.3 |
| 20 | Chlorogenic acid | 1794427 | C16H18O9 | -7.2 |
| 21 | Erythraline | 5317205 | C18H19NO3 | -7 |
| 22 | Osthole | 10228 | C15H16O3 | -6.9 |
| 23 | AC-TYR(AC)-OH | 87072 | C13H15NO5 | -6.7 |
| 24 | Adenosine | 60961 | C10H13N5O4 | -6.1 |
| 25 | Vanillyl mandelic acid | 1245 | C9H10O5 | -6.1 |
| 26 | Ferulic acid | 445858 | C10H10O4 | -6.1 |
| 27 | P-coumaric acid | 637542 | C9H8O3 | -5.9 |
| 28 | Uridine | 6029 | C9H12N2O6 | -5.9 |
| 29 | Protocatechuic acid | 72 | C7H6O4 | -5.7 |
| 30 | Protocatechualdehyde | 8768 | C7H6O3 | -5.7 |
| 31 | Gallic acid | 370 | C7H6O5 | -5.6 |
| 32 | Quinic acid | 516576382 | C7H12O6 | -5.6 |
| 33 | Coumarin | 323 | C9H6O2 | -5.6 |
| 34 | L-Phenylalanine | 6140 | C9H11NO2 | -5.5 |
| 35 | Gentisic acid | 3469 | C7H6O4 | -5.4 |
| 36 | Nicotinamide | 936 | C6H6N2O | -5.2 |
| 37 | Vanillin | 1183 | C8H8O3 | -5.1 |
| 38 | Adenine | 190 | C5H5N5 | -5.1 |
| 39 | p-Hydroxybenzaldehyde | 126 | C7H6O2 | -5 |
| 40 | Stachydrine | 115244 | C7H13NO2 | -4.9 |

**Table S4. Binding energy of molecular docking for ROCK1(Cont.)**

| Number | Monomer | Pubchem Number | Molecular Formula | Vina |
| --- | --- | --- | --- | --- |
| 41 | Malic acid | 525 | C4H6O5 | -4.9 |
| 42 | Linoleic acid | 5280450 | C18H32O2 | -4.9 |
| 43 | 1-Linoleoyl-sn-glycero-3-phosphocholine | 56955896 | C27H52NO7P | -4.8 |
| 44 | Citric acid | 311 | C6H8O7 | -4.8 |
| 45 | Mannitol | 6251 | C6H14O6 | -4.7 |
| 46 | Methyl linoleate | 5284421 | C19H34O2 | -4.7 |
| 47 | D-(-)-Tartaric Acid Diethyl Ester | 117410 | C8H14O6 | -4.5 |
| 48 | Succinic acid | 1110 | C4H6O4 | -4.5 |
| 49 | Linoleoyl ethanolamide | 5283446 | C20H37NO2 | -4.4 |
| 50 | 5-Hydroxymethylfurfural | 237332 | C6H6O3 | -4.2 |
